# Large genomic differences between the morphologically indistinguishable diplomonads *Spironucleus barkhanus *and *Spironucleus salmonicida*

**DOI:** 10.1186/1471-2164-11-258

**Published:** 2010-04-21

**Authors:** Katarina Roxström-Lindquist, Jon Jerlström-Hultqvist, Anders Jørgensen, Karin Troell, Staffan G Svärd, Jan O Andersson

**Affiliations:** 1 Department of Cell and Molecular Biology, Uppsala University, Uppsala, Sweden; 2 Department of Parasitology, National Veterinary Institute, Oslo, Norway; 3 Department of Evolution, Genomics and Systematics, Uppsala University, Uppsala, Sweden

## Abstract

**Background:**

Microbial eukaryotes show large variations in genome structure and content between lineages, indicating extensive flexibility over evolutionary timescales. Here we address the tempo and mode of such changes within diplomonads, flagellated protists with two nuclei found in oxygen-poor environments. Approximately 5,000 expressed sequence tag (EST) sequences were generated from the fish commensal *Spironucleus barkhanus *and compared to sequences from the morphologically indistinguishable fish parasite *Spironucleus salmonicida*, and other diplomonads. The ESTs were complemented with sequence variation studies in selected genes and genome size determinations.

**Results:**

Many genes detected in *S. barkhanus *and *S. salmonicida *are absent in the human parasite *Giardia intestinalis*, the most intensively studied diplomonad. For example, these fish diplomonads show an extended metabolic repertoire and are able to incorporate selenocysteine into proteins. The codon usage is altered in *S. barkhanus *compared to *S. salmonicida*. Sequence variations were found between individual *S. barkhanus *ESTs for many, but not all, protein coding genes. Conversely, no allelic variation was found in a previous genome survey of *S. salmonicida*. This difference was confirmed by sequencing of genomic DNA. Up to five alleles were identified for the cloned *S. barkhanus *genes, and at least nineteen highly expressed *S. barkhanus *genes are represented by more than four alleles in the EST dataset. This could be explained by the presence of a non-clonal *S. barkhanus *population in the culture, by a ploidy above four, or by duplications of parts of the genome. Indeed, genome size estimations using flow cytometry indicated similar haploid genome sizes in *S. salmonicida *and *G. intestinalis *(~12 Mb), whereas the *S. barkhanus *genome is larger (~18 Mb).

**Conclusions:**

This study indicates extensive divergent genome evolution within diplomonads. Genomic traits such as codon usage, frequency of allelic sequence variation, and genome size have changed considerably between *S. barkhanus *and *S. salmonicida*. These observations suggest that large genomic differences may accumulate in morphologically indistinguishable eukaryotic microbes.

## Background

Eukaryotic genomes are very diverse in size, content, structure and mode of inheritance. The size of haploid genomes varies extensively. Some microsporidia have genomes of a few mega base pairs (Mb), whereas amoeba, plants and animal genomes can be thousands of Mb [1]. There are also large differences in genome organization within and between eukaryotic groups [1]. The frequency and occurrence of meiotic sex also varies extensively between eukaryotic lineages, even if it is problematic to determine whether a specific lineage undergoes meiosis or other kinds of genetic recombination [2]. Very little is known about the variation of these various traits within and between different eukaryotic groups except for a selection of extensively studied fungi, plants and animals. We are currently performing a comparative genomics project on representatives from the microbial eukaryotic group Diplomonadida (diplomonads), with the overall aim to deepening the understanding of the factors that shape eukaryotic genomes in general, and diplomonad genomes in particular.

Diplomonads are a group of anaerobic, or micro-aerophilic, flagellated protists classified within Excavata [3]. All members of the group, with the exception of the monokaryotic enteromonads, have double sets of nuclei, flagella and other organelles [4]. Diplomonads are frequently found in environments depleted with oxygen, and the group contains pathogens, commensals and free-living species [5]. The intestinal parasite *Giardia intestinalis *(syn. *G. lamblia *and *G. duodenalis*), a major cause of waterborne enteric disease in humans, is the most studied diplomonad [6-8]. There are currently seven different genotypes (A-G) identified within the *G. intestinalis *species complex; human infections are caused by genotypes A and B [6]. However, it has been suggested that the morphological species *G. intestinalis *should possibly be divided into several species based on host specificities and genetic differences [6,7]. The determination of the true phylogenetic relationships within the diplomonads is difficult. Free-living *Hexamita *and *Trepomonas *are nested among pathogenic members of *Spironucleus*. Furthermore, enteromonads, are nested deep within classical diplomonads in molecular phylogenies, suggesting that these are secondarily monokaryotic, or that the diplokaryotic state have multiple origins within diplomonads [4,8,9].

In this study we focus on *Spironucleus barkhanus *and *Spironucleus salmonicida*. Both species were previously known as *S. barkhanus*. However, parasites causing systemic infections in Atlantic salmon *Salmo salar*, Chinook salmon *Oncorhynchus tshawytscha *and Arctic char *Salvelinus alpinus *[10], are now classified as *S. salmonicida *[11]. These parasites have caused severe problems for fisheries [12,13]. The closely related *S. barkhanus *is a commensal in wild freshwater populations of Arctic char and grayling *Thymallus thymallus *[11]. These morphologically indistinguishable organisms are defined as separate species on the basis of their ecology (commensal and parasite) and that they form two clades in gene trees [11]. The genetic divergence is between 8% and 30% for the ribosomal RNA, alpha-tubulin and glutamate dehydrogenase genes [11]. The ATCC 50377 strain of *S. salmomicida *is the most studied diplomonad on the genetic level outside the *Giardia *genus [14], although there is an ongoing genome project on *Spironucleus vortens *[15].

The genome projects on *S. salmonicida *and *G. intestinalis *revealed reduced and compact genomes with few, if any, genes with introns [14,16]. There are also variations between the diplomonad genomes. A number of lineage-specific genes obtained from bacteria were detected in *S. salmonicida *and the codon usages are drastically different [14]. In addition, all hexamitinid diplomonads (including *Spironucleus*) utilize an alternative genetic code, whereas the canonical code is used by members of the *Giardia *genus [4,17] The level of sequence divergence within diplomonad cells also vary extensively. Both nuclei in the vegetative trophozoite stage of *G. intestinalis *are diploid [18]. *In situ *hybridization studies of *G. intestinalis *genotype A have shown that each nucleus contains at least one complete copy of the genome and that the two nuclei are partitioned equationally at cytokinesis [19]. Allelic variations are expected to accumulate in the absence of genetic exchange between the two nuclei [20]. Nevertheless, a very low frequency of sequence heterozygosity is present in the *G. intestinalis *genotype A (WB) genome [21] and no allelic sequence variation was reported from the *S. salmonicida *genome [14]. Genetic exchange might occur between the nuclei, suggesting a mechanism that maintains a low sequence divergence [22]. However, the efficiency of such a mechanism appears to vary between closely related lineages; the draft *G. intestinalis *GS (genotype B) genome revealed an overall allelic sequence divergence of 0.5% [7].

We have performed a comparative genomic project on *S. barkhanus *and *S. salmonicida*, including the generation of ~5000 expressed sequence tag (EST) sequences from *S. barkhanus *and an estimation of genome sizes of both species. The goal was to increase the understanding of the tempo and mode of genomic structure and content evolution within the diplomonads. The present study present several unexpected observations such as large differences of diplomonad genome size and codon usage, and a high level of allelic sequence variation in *S. barkhanus*.

## Results and Discussion

*S. barkhanus *and *S. salmonicida *are two morphologically indistinguishable protists which have recently been classified as two distinct species based on ecological and genetic data from a few genes [11]. We isolated *S. barkhanus *from the wild freshwater salmonid grayling (*Thymallus thymallus*) to further study the genetic differences between these two diplomonads. *In vitro *growth in TYI-S-33 medium was successfully established for both this new isolate and *S. salmonicida *(ATCC 50377), although, the optimal growth conditions differed. The generation time for *S. barkhanus *at 4°C in the presence of bile was approximately the same as the generation time for *S. salmonicida *at 15°C without bile. The morphology is indistinguishable (Additional files 1-2) and the six anterior flagella contribute to the high speed swimming, even at 4°C (Additional files 3-4).

### Origin of *Spironucleus *gene repertoire

We constructed a CloneMiner cDNA library from RNA harvested from *S. barkhanus *(see Method section for details). Approximately 5,000 clones were randomly picked and sequenced. The obtained chromatograms were clustered into 1,270 unique sequences. 831 of these showed similarity with E values less than 1e^-5 ^to previously known protein-coding genes outside the *Spironuclues *genus (Additional file 5). Putative orthologs were found for 233 of these among the sequences from the *S. salmonicida *genome survey [14]. The average identities were 84% and 76% on the amino acid and nucleotide levels, respectively. This is in the similar range as orthologous genes between the morphologically identical *G. intestinalis *genotype A (WB) and B (GS) isolates [7], indicating large genetic divergence between morphologically similar diplomonad species.

We divided the identified protein coding genes into three classes based on their similarities to database sequences (Additional file 5). The first class contain 538 proteins with highest similarities to *Giardia *proteins [21], strongly suggesting these to be present in the ancestral diplomonad cell. This class contains proteins performing basic functions in eukaryotic cells such as general metabolism, translation, flagellar function and chromatin structure. However, proteins performing functions with a more limited distribution among eukaryotes are also included. An enzyme involved in cyst-wall synthesis, two enzymes in the arginine dihydrolase pathway which enable the diplomonads to utilize arginine as an energy source under limited oxygen conditions [23,24], and A-type flavoproteins which are widespread in anaerobic protists, but rare in other eukaryotes [25] are all members of this class.

A second class consists of 235 genes with homologs present in the *Giardia *genome, but which show higher similarities to homologs from organisms outside diplomonads. Many of these probably represent divergent sequences of genes present in the common diplomonad ancestor; most of them show almost as high similarity to *Giardia *genes. A number of ribosomal and proteasomal proteins are present in this class. Interestingly, 59 of the 71 sequences with a cysteine content of the putative coding region of more than 10% (Additional file 5) belong to this class. Many show highest similarities to cysteine-rich proteins in ciliates, and the conserved motifs of *G. intestinalis *variant surface proteins (CRGKA and GGCY [26]) could not be found. This indicates a high sequence divergence of cysteine-rich proteins within diplomonads, as previously observed in the *S. salmonicida *genome survey [14]. Indeed, these protein families were the most divergent between *G. intestinalis *WB and GS [7]. Annexins is another protein family that is very divergent within diplomonads. Alpha-giardins are annexin-like proteins in a 20 member *Giardia*-specific protein family associated with different cytoskeleton and membrane structures [27]. All annexin-like proteins in *Spironucleus *are more similar to annexins in other eukaryotes than to any alpha-giardin. For example, the putative amino acid sequence of contig160 shows 40% identity to *Xenopus laevis *annexin A7, but only 27% identity to *G. intestinalis *alpha-5 giardin. The most parsimonious explanation probably is that alpha-giardins evolved specifically in the *Giardia *branch from typical eukaryotic annexins. However, an alternative explanation for the large divergence of annexin-like proteins in diplomonads is that one of the two studied lineages obtained the proteins via lateral gene transfer.

**Figure 1:**
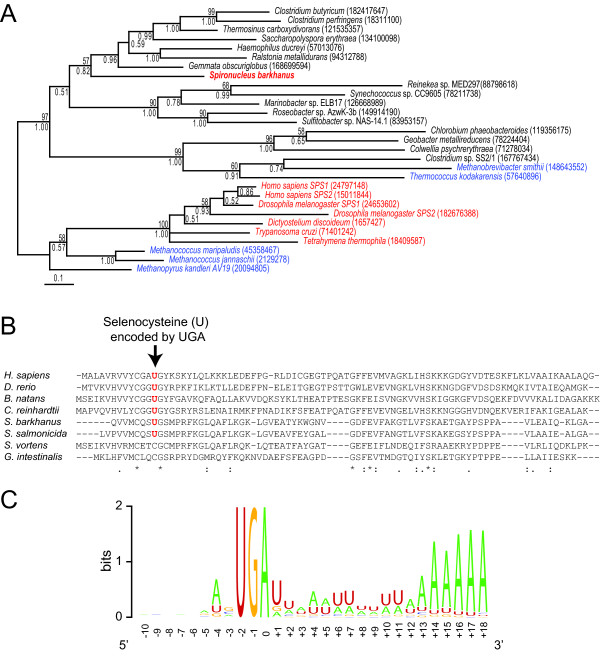
**UGA serves as a codon for selenocysteine and a polyadenylation signal in addition to termination in *S. barkhanus *and *S. salmonicida***. (A) Phylogenetic tree of putative *S. barkhanus *selenophosphate synthetase with homologs representing the diversity of the protein family. Eukaryotes, Archaea and Bacteria are shown in red, blue and black, respectively. Maximum likelihood bootstrap values and bayesian posterior probabilities are shown above and below branches, respectively. GenBank identification numbers (gi) for each sequence are shown in parentheses. (B) Alignment of a putative *S. barkhanus *selenoprotein W1 (contig253) with homologs from diplomonads (*S. salmonicida *- gi:120476942, *G. lamblia *- gi:253741748, and *S. vortens *[15]), and a selection of other eukaryotes (*Homo sapiens *- gi:4506887, *Danio rerio *- gi:29648542, *Chlamydomonas reinhardtii *- gi:159471514, *Bigelowiella natans *- gi:47028259). (C) Sequence logo [54] around the termination codon of 134 *S. barkhanus *cDNA sequences.

The third class of genes consists of 58 sequences without matches in the *G. intestinalis *genome (Additional files 5 and 6). Metabolic functions dominate among the 26 that do have putative annotations, including several peptidases, desulfoferredoxin, fructokinase, cartenoid isomerase and rubrerythrin (Additional file 6), suggesting metabolic adaptation as a selection force for their maintenance in the *S. barkhanus *genome. Detailed phylogenetic analyses, such as for selenophosphate synthetase (Figure 1A), would be necessary to determine the origin of these genes; they have either been gained in the *Spironucleus *lineage or been lost in the *Giardia *lineage. Lateral gene transfer has indeed previously been shown to contribute to adaptation within diplomonads and other eukaryotes [7,14,21,28-30]. In fact, ten of the *S. barkhanus *sequences have indeed close homologs among proteins identified as recently introduced via lateral gene transfer into the *S. salmonicida *genome (Additional file 6). Several of the other proteins also likely represent acquisitions in the *Spironucleus *lineage, some maybe after the divergence between *S. barkhanus *and *S. salmonicida*. At any rate, this class contains candidate genes for the understanding of the diversifications of diplomonads, regardless of their origins, as exemplified below.

### A triple function of UGA as sole termination codon, selenocysteine codon and polyadenylation signal

*Spironucleus *uses an alternative genetic code; UAA and UAG encode glutamine, whereas UGA is the sole termination codon [17]. A putative selenocysteine tRNA (SelC) was identified in the *S. salmonicida *genome indicating that all 64 codons might be used to encode amino acids in this organism [14]. Here we discovered a putative selenophosphate synthetase (SelD), an enzyme marker for selenium utilization [31]. A phylogenetic analysis shows the *S. barkhanus *homolog nested within bacterial SelD sequences (Figure 1A). Furthermore, a *S. salmonicida *EST with 40% identity over 238 amino acids to ciliate selenocysteine (Sec)-specific elongation factor (SelB) was identified (gi: 120477278). Thus, three gene signatures (SelB, SelC and SelD) for the Sec-decoding trait [31] have been identified in *S. salmonicida *or *S. barkhanus*. A weak similarity to selenoprotein W1, a previously identified selenoprotein, was found within our dataset (Figure 1B). A UGA codon is found in the *S. barkhanus *and *S. salmonicida *homologs of selenoprotein W1 in the amino acid position where a selenocysteine is incorporated in other eukaryotes (Figure 1B). Together, these observations strongly suggest that these two diplomonads are able to incorporate Sec into proteins using the UGA codon. It is unknown how the translation machinery distinguish between termination and Sec-insertion in *S. barkhanus*; no canonical Sec-insertion sequences (SECIS) could be detected within our dataset using ERPIN [32,33].

No selenium utilization trait has been reported from *Giardia *to our knowledge. Indeed, no genes coding for homologs to SelB or SelD could be found in any of the available *G. intestinalis *genomes [7,21], nor in the released sequences form the *S. vortens *project [15]. Interestingly, these two species encode cysteine in the homologous position of the selenocysteine in selenoprotein W1 (Figure 1B). Exchanges between Cys and Sec within selenoproteins are frequent in evolution [34]. These observations indicate that *G. intestinalis *and *S. vortens *lack the Sec-decoding trait. If so, Sec-decoding capacity has either been lost independently in the *S. vortens *and *G. intestinalis *lineages, or been gained in the lineage leading to *S. barkhanus *and *S. salmonicida*. Both gains and losses of the trait are common in bacterial evolution [34]. Interestingly, the position of *S. barkhanus *selenophosphate synthetase nested within prokaryotic sequences in the phylogenetic tree (Figure 1A) indicates a recent acquisition of that gene. Furthermore, no homologs of SelB or SelD could be identified in *Trichomonas vaginalis*, the nearest neighbour of diplomonads with an available genome sequence [35]. These findings circumstantially support a recent gain of the selenium utilization trait in the ancestor of *S. barkhanus *and *S. salmonicida*.

UGA probably also function as a polyadenylation signal in *S. barkhanus *and *S. salmonicida*. The positions of the polyA tail were mapped in 134 of the clustered sequences. The 3' untranslated regions were found to be short, around 13 bp (Figure 1C). This is similar to the other studied diplomonads [14,36], but unlike other eukaryotes [37]. The conservation pattern is very similar between *S. barkhanus *and *S. salmonicida*, with a strong positional correlation between the termination codon and the beginning of the polyA-tail, but only weak conservation outside the UGA codon, the only functional termination codon in *Spironucleus *[17]. In both organisms there is a preference for A in the position two bases upstream of the termination codon and U immediately downstream (Figure 1C) [14]. In other studied eukaryotes, the distance between the polyadenylation signal and the polyA site is conserved, whereas 3' untranslated regions vary in length [37]. The positional correlation between the termination codon and the polyA site in *S. barkhanus *(Figure 1C) and *S. salmonicida *[14] suggests that the single termination codon [17] likely also function as a polyA signal, in addition to the function as a Sec-codon in selenoproteins. Interestingly, a eukaryotic peptide chain release factor subunit 1 that recognizes stop codons and terminates translation was also identified in this survey (Additional file 5), which will make it possible to study the interaction between polyadenylation, translational termination and selenocysteine incorporation.

### A drastic shift in codon usage between the genomes

Several codons may code for the same amino acid in protein coding genes due to the degenerate nature of the genetic code. The main determinant for codon-usage lineage-variations among genomes often is genome-wide mutational processes, whereas selection typically is invoked to explain differences within genomes [38,39]. The codon usage of *G. intestinalis *can be explained by a combination of these forces where a subset of codons are preferred in highly expressed genes [14,40]. The genome survey of *S. salmonicida *revealed a similar pattern for the majority of the genes [14]. However, a minority of the *S. salmonicida *genes showed codon-usage patterns with high GC3_s _values close to 1 although the genome in general is G+C poor. These observations were very difficult to explain using the traditional interpretations [14,38,39]. Here we take a comparative approach to study the codon usage in *Spironucleus*.

**Figure 2:**
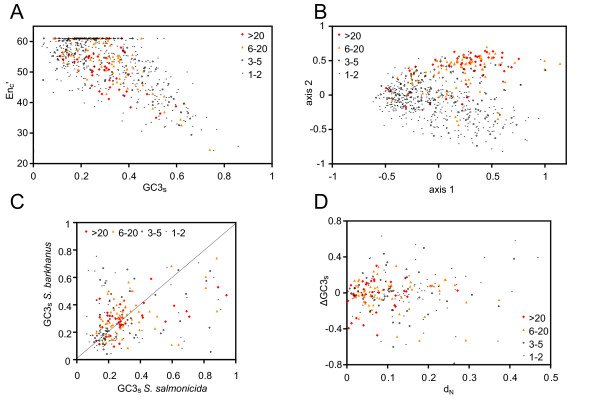
**Comparison of codon usage in *S. barkhanus *and *S. salmonicida***. (A) The effective number of codons (N_c_') plotted against the G+C content in four-fold degenerate positions in the gene (GC3_s_) for 682 unique *S. barkhanus *genes. (B) Correspondence analysis of the relative synonymous codon usage (RSCU) of the 682 unique *S. barkhanus *genes. (C) GC3_s _of aligned regions of 233 pairs of putative *S. barkhanus *and *S. salmonicida *orthologs. (D) The difference of GC3_s _of aligned regions of putative orthologs plotted against the non-synonymous divergence values (d_N_). The color coding indicates the number of times the gene was sampled within the *S. barkhanus *EST data.

*S. barkhanus *has a relatively G+C-poor genome; the average G+C-content of the EST sequences is 41% and GC3_s _values below 40% for most genes (Figure 2A), suggesting a general mutational bias towards A+T. We classify genes as "highly expressed" and "weakly expressed" if they have been found more than 20 times or less than 3 times, respectively, among the clones from the cDNA library. The number of occurrences of a specific gene within a non-normalized cDNA library is expected to be roughly correlated with the amount of mRNA in the cells harvested for the library preparation. Specific codons are preferred in highly expressed *S. barkhanus *genes, as previously found in *S. salmonicida *[14] (data not shown). The codon usage was explored in more detail by plotting the effective number of codons (En_c_') [41] against the GC3_s _values (Figure 2A) and by correspondence analyses on the relative synonymous codon usage (Figure 2B). The *S. barkhanus *GC3_s _values are weakly correlated with expression levels (Figure 2A), whereas the correspondence analysis clearly separates highly expressed genes from weakly expressed genes (Figure 2B). This is in agreement with the observation that the preferred codons are a mixture of G+C rich and G+C poor codons [14]. Overall, the *S. barkhanus *codon usage is similar to *S. salmonicida*, except that the variation in GC3_s _values appear less extreme in *S. barkhanus *[14] (Figure 2A).

This aspect of codon usage variation between *S. barkhanus *and *S. salmonicida *was studied in more detail by extracting homologous regions of 233 putatively orthologous genes from the two datasets. The *S. barkhanus *orthologs of *S. salmonicida *genes with unusually high GC3_s _values tend to have lower GC3_s _values (Figure 2C). In *S. salmonicida*, 14 genes show GC3_s _values above 75%, whereas none of their putative *S. barkhanus *orthologs do (Figure 2C). We plotted the difference between the putative orthologs against the frequency of non-synonymous changes in the gene (d_N_), which is a measure of divergence, to examine whether the genes that show a drastic shift in GC3_s _values tend to represent divergent genes (Figure 2D). Many of the genes with large differences in GC3_s _show small non-synonymous divergences. Thus, the drastic difference between the two genomes is unlikely due to erroneous assignments of orthologs. These observations suggest that divergent codon usages has emerged rather recently in a subset of the *S. salmonicida *genes [14], or been lost in the *S. barkhanus *lineage. *S. salmonicida *genes with extremely high GC3s values were correlated with genomic regions with unusually high G+C content [14]. Our data indicate that these genomic regions are less pronounced in the *S. barkhanus *genome.

### Sequence heterogeneity between *S. barkhanus *alleles

Many *S. barkhanus *sequences show high identity within the dataset. For example, there are seven sequences annotated as alpha-tubulin. Using a BLASTN E value cutoff of 1e^-20 ^the 1,270 unique sequences were divided into 1,097 groups consisting of 1 to 7 sequences. 107 of these consisted of two or more sequences (Additional file 5). This indicates that *S. barkhanus *encodes a large number of alleles and/or paralogs. Manual inspection of clustered sequences revealed heterogeneity also between aligned EST sequences indicating the occurrence of single nucleotide polymorphisms (SNPs). One or more SNPs were identified in 166 of the 1270 sequences, using the Polybayes software [42] (Additional file 5). These results were surprising given that the genomic survey of *S. salmonicida *failed to indicate any allelic sequence variation [14]. There are several possible origins of the SNPs detected in the *S. barkhanus *dataset. They could represent alleles present within a single *S. barkhanus *lineage and/or alleles from different *S. barkhanus *lineages present in the grayling and maintained during 13 passages of *in vitro *culture. In principle, they could also be cloning artefacts produced during the preparation of the EST library. We performed additional experiments and analyses to study the phenomenon in more detail and to distinguish between these alternatives.

The genes encoding enolase, ribosomal protein S2, glutamate dehydrogenase, heat shock protein 70 and pyruvate kinase were selected for cloning experiments. All are single-copy genes in the *G. intestinalis *genome [21] and show varying frequencies of SNPs in the *S. barkhanus *EST sequences (Table 1). Primers were designed that amplified ~500 bp regions in each gene from *S. barkhanus *and *S. salmonicida *genomic DNA. The PCR products were cloned and between 10 and 68 individual clones were sequenced from each amplified region. The cloning experiments identified all but two of the 20 SNPs identified in the *S. barkhanus *ESTs with the Polybayes software, all with the same variation (Table 1). An additional ten SNPs not found in the *S. barkhanus *EST data were identified in the cloning experiments (Table 1). All were detected in multiple clones, except one only found in a single clone of heat shock protein 70. The absence of these SNPs in the EST data is likely due to poor coverage, possibly in combination with differential expression of alternative alleles. Indeed, five of the novel SNPs were found in the glutamate dehydrogenase, a gene represented by very few EST clones (Table 1). These results strongly suggest that the variation in the EST library corresponds to sequence variation present in the genomic DNA, showing that the SNPs identified in the EST data are not due to cloning artefacts.

**Table 1:** PCR amplifications of regions with SNPs observed in the EST data.

*Gene*	***species***^**a**^	*PCR*		*#SNP*			*#alleles PCR*		*#clones major and (minor) alleles*
		*length*	*#clones*	*PCR*	*EST*	*shared*	*total*	***major***^**b**^	
Enolase	SB	566	14	4	4	4	4	3	6,4,3 (1)
Enolase	SS	566	10	0	0	0	1	1	10
Ribosomal protein S2	SB	496	10	0	0	0	1	1	10
Ribosomal protein S2	SS	499	10	0	0	0	1	1	10
Glutamate dehydrogenase	SB	549	13	6	2	1	2	2	9,4
Glutamate dehydrogenase	SS	549	13	10	N/A^c^	N/A^c^	3	2	8,4 (1)
HSP70	SB	514	67^d^	5	5	4	6	5	54,5,4,2,1 (1)
HSP70	SS	514	10	0	N/A^c^	N/A^c^	1	1	10
Pyruvate kinase	SB	466	68^d^	13	9	9	11	3	43,8,6 (2,2,2,1,1,1,1,1)
Pyruvate kinase	SS	466	10	0	0	0	1	1	10

^a^) SB: *Spironucleus barkhanus*, SS: *Spironucleus salmonicida*

^b^) Major alleles are the minimal set of alleles observed in the PCR experiments to explain all SNPs present in the data.

^c^) No *S. salmonicida *ESTs were available for these two genes.

^d^) From two independent PCR reactions.

Interestingly, the SNPs are not evenly distributed among the genes selected for the cloning experiments. Four *S. barkhanus *genes have four or more SNPs, whereas none was found in the ribosomal protein S2 (Table 1). The picture is similar in the EST data; some *S. barkhanus *genes lack SNPs, whereas others have large numbers (Additional file 5). This variation does not appear to be correlated with the expected level of conservation. For example, ribosomal protein L13 is present in several highly similar contigs in the EST assembly, each containing SNPs, whereas ribosomal protein S2 only has a single allele (Table 1 and Additional file 5). There are several plausible explanations for these observations. In principle, the sequence variation could originate from clonal *S. barkhanus *lineages present in the fish and then maintained in the culture. If so, the level of variation would be expected to be relatively evenly distributed among the genes with the most conserved genes showing the least variation. This does not seem to be the case (Table 1 and Additional file 5). However, the observed pattern could originate from sexual recombination between two different lineages followed by autogamy that purges most of the allelic variation from each lineage, suggesting the presence of at least two lineages in the culture. Yet another explanation is that the sequence variation represents allelic differences within a single clone, or very closely related lineages, of *S. barkhanus*. In this case the level of variation could vary considerably between genes due to recent local within-cell recombination that removes allelic variation. We tend to favour the latter model, mainly because it agrees with the patterns observed in the genomes of *G. intestinalis *GS [7], and *S. vortens *(see below). However, additional data is clearly needed to distinguish between the alternatives.

### The degree of sequence variation differs between *Spironucleus *species

Comparison of sequences from individual clones from PCR reactions revealed between one and eleven alleles for the five amplified *S. barkhanus *genes (Table 1), which is puzzling if they are assumed to come from a single lineage. Closer examination of the occurrence of SNPs among the alleles showed that all SNPs often were represented in a subset of the alleles. For example, the thirteen SNPs in *S. barkhanus *pyruvate kinase were represented in three alleles. The other nine alleles are different combinations of these three major alleles (Additional file 7). 57 of the 68 clones represent these major alleles (Table 1). It could be that the minor alleles are artefacts of the cloning and sequencing procedure; chimeras are expected to occur in amplifications of closely related sequences [43]. Using this rationale, the PCR experiments identified between one and five major alleles for the five cloned *S. barkhanus *genes (Table 1). Duplication of a segment in the *S barkhanus *genome, followed by divergence could result in more than four distinct sequences of alleles and paralogs, even if the organism is tetraploid.

Ten SNPs were found in the *S. salmonicida *glutamate dehydrogenase gene, whereas none were found in any of the other four *S. salmonicida *genes in the cloning experiments (Table 1). The presence of SNPs in glutamate dehydrogenase is probably due to a gene duplication followed by divergence; our preliminary assembly of ~4× coverage of the *S. salmonicida *genome indicates distinct upstream sequences for two paralogs (unpublished data). Thus, our study indicates a low level of allelic sequence divergence in *S. salmonicida*, as suggested by the previous genomic survey [14].

Available sequences from the genome project of *S. vortens *[44] were used to test whether allelic variation is present in this *Spironucleus *species. Sequences covering the homologous region of the five genes were identified among the >200,000 genomic survey sequences (GSS) and >25,000 EST sequences available at the NCBI [45]. No sequences were found for glutamate dehydrogenase, whereas between five and 32 GSSs, and between nine and hundreds of ESTs were found for the other four genes (Table 2). The number of identified SNPs varied extensively between the four genes; none were found for the enolase gene, whereas more than 60 SNPs were present in the pyruvate kinase sequences (Table 2). The number of alleles appeared to be high for the three genes for which SNPs were found (data not shown). Thus, extensive allelic sequence variation is present in *S. vortens*.

**Table 2:** SNPs observed in homologous regions in the *S. vortens *genome.

*Gene*	*length*	***#GSS***^**a**^	***#EST***^**a**^	*#SNP*
Enolase	563	5	9	0
Ribosomal protein S2	508	5	>300	10
Glutamate dehydrogenase	N/A^b^	-	-	-
HSP70	517	24	~100	>40
Pyruvate kinase	451	32	>300	>60

^a^) Covering at least 80% of the region.

^b^) Not available; no glutamate dehydrogenase sequences were detected in the released *S. vortens *sequences.

### An emerging picture of allelic sequence variation in diplomonads

Our data indicate large variations in the degree of allelic sequence variation between different genes and different member of the genus *Spironucleus*. This is similar to the situation in *G. intestinalis*; WB (genotype A) and GS (genotype B) showed <0.01% and 0.5% variations, respectively [7,21]. Furthermore, *G. intestinalis *genotype B isolates repeatedly show higher frequencies of double-peaks in sequence chromatograms from PCR amplifications from patient samples in epidemiological studies of *Giardia *than genotype A isolates [46,47]. The allelic sequence divergence in *G. intestinalis *GS is non-randomly distributed along the chromosomes with large regions with very low frequencies of SNPs followed by large regions with high divergence [7]. Similarly, some *Spironucleus *genes lack SNPs, whereas others have large numbers (Tables 1, 2 and Additional file 5).

**Figure 3:**
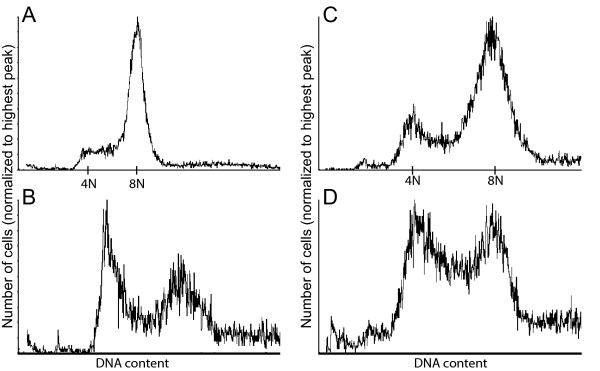
**Flow cytometry analysis of genomic size of *S. barkhanus *and *S. salmonicida***. (A) and (C) Flow-cytometric analysis of the total DNA content of exponentially growing *G. intestinalis *WB trophozoites. The G1 and G2/M peaks are labeled with 4N and 8N, corresponding to a total genome content of 48 and 96 Mbp. (B) Flow-cytometric analysis of the total DNA content of exponentially growing *S. barkhanus *trophozoites. (D) Flow-cytometric analysis of the total DNA content of exponentially growing *S. salmonicida *trophozoites.

In *G. intestinalis *GS most genes with SNPs seem to group into two, or sometimes three alleles [7]. Our data suggest higher numbers of alleles for many of the *S. barkhanus *genes (Table 1 and Additional file 5). Ten proteins are represented by five or more closely related sequences which correspond to alleles and/or paralogs. An additional nine proteins with three or four highly similar contig sequences show allelic variation within individual clusters suggesting that also these have five or more alleles. A similar picture emerges from the limited analyses of *S. vortens *data (Table 2). The genome project of *S. vortens *reported large difficulties with genome assembly [15]. Total scaffold and contig lengths were 104 and 33 Mb, respectively, which is much larger than the previously estimated genome size of 16 Mb [44]. A probable cause of the problems is the presence of extensive allelic variation for a large part of the genome. Local gene duplications followed by divergence may also contribute to the high allelic numbers found in *S. vortens *and *S. barkhanus*. Interestingly, the *S. vortens *genes with the highest number of SNPs in our study have the largest number of GSSs (Table 2), circumstantially suggesting duplications of these genes. Comparative genome size estimates of *S. barkhanus *and *S. salmonicida *were performed to test whether the differences in allelic sequence variation were correlated with genome size variations (Figure 3 and Additional file 8).

### Divergent genome sizes of *S. barkhanus *and *S. salmonicida*

We studied the genome size using flow cytometry analysis of *S. barkhanus *and *S. salmonicida *cells with fluorescently labelled DNA (Figure 3). The total amount of DNA in each cell was compared to the amount of DNA in *G. intestinalis *WB trophozoites, which has been estimated to have a haploid genome size of 12 Mb [48]. Exponentially growing *Giardia *trophozoites display two peaks with cells in the G1 and G2/M phases of the cell cycle with ploidies of 4N and 8N, respectively. The majority of cells can be found in the G2/M phase [18]. The G1 peak corresponds to a total genome size of 48 Mb (4 × 12 Mb) and the G2/M peak to a genome size of 96 Mb (8 × 12 Mb) (Figure 3A and 3C). *S. barkhanus *has two major peaks that correspond to total genome sizes of around 72 and 144 Mb (Figure 3B). This corresponds to a haploid genome size of ~18 Mb, if a cycling of ploidy between 4N and 8N is assumed. The major peaks of *S. salmonicida *are very close to the peaks of *G. intestinalis*, suggesting a haploid genome size around 12 Mb for *S. salmonicida *(Figure 3CD).

We also studied the genome size using pulsed-field gel electrophoresis (PFGE). Unfortunately, the chromosomes were poorly separated in our PFGE experiments, preventing precise estimations of genome sizes using this method (Additional file 8). Nevertheless, our genome size determinations using flow cytometry show a large difference in haploid genome sizes between *S. barkhanus *and *S. salmonicida*. This suggests that genome sizes are dynamic within diplomonads and may differ considerably even between relatively closely related and morphological indistinguishable species.

## Conclusions

We have performed a comparative study of *S. barkhanus *and *S. salmonicida*, as a part of our ongoing project on comparative genomics in diplomonads. Traits which distinguish these from other studied diplomonads were identified, such as the ability to incorporate selenocysteine into proteins. Our results also indicate that the genome sizes differ drastically between the two morphologically indistinguishable *Spironucleus *species. There are also differences in codon usages and the occurrence of allelic sequence variation. A large number of *S. barkhanus *genes have high frequencies of SNPs, whereas *S. salmonicida *genes show sequence homogeneity. The presence and absence of SNPs among genes and genomes are the results of interplay of mutations and recombinatory events within and between isolates. In *G. intestinalis*, the presence of meiotic sex has been suggested [49], the nuclei have been suggested to meet in the cyst with the possibility of genetic exchange [22], and exchange of genetic material between natural isolates has been described [50]. Our data suggest that the outcome of these different putative processes may be rather different in closely related diplomonads. Further studies are obviously needed to understand the importance of the various putative phenomena acting on diplomonad genomes. The observations in this study are in agreement with our studies of *G. intestinalis *genomes [7]. Thus, large genomic differences between morphologically indistinguishable isolates are widespread among diplomonads.

## Methods

### Organisms and cultures

Trophozoites of *S. barkhanus *were isolated from the gall bladder of the freshwater salmonid grayling in Glomma River, south-eastern Norway. An axenic culture of *S. barkhanus *was grown at 4°C in TYI-S-33 medium supplemented with bile according to Keister [51]. The identity of the isolate was verified by sequencing of the small subunit ribosomal RNA gene PCR amplified from the genome [52]. *S. salmonicida *(ATCC 50377), isolated from a muscle abscess in Atlantic salmon Vesteraalen, northern Norway (previously known as *S. barkhanus *[11]) was obtained from American Type Culture Collection (ATCC) and grown in axenic culture following the ATCC protocol.

### RNA isolation, cDNA library construction and sequencing

Exponentially growing *S. barkhanus *were harvested at passage 13. After collection by centrifugation (5 min at 2000 rpm, 4°C) the cells were directly lysed in Trizol reagent (Invitrogen) and total RNA was isolated according to the manufacturer's instruction. The amount and quality were analyzed by nanodrop and formaldehyde-denaturing 1% agarose gel before total RNA was ethanol precipitated. Following the isolation of mRNA using Poly(A)PuristTM MAG system (Ambion), directed and size-fractionated cDNA libraries was made using the CloneMinerTM cDNA Library Construction Kit (Invitrogen). Positive transformants were manually picked for sequencing. Sequencing template preparation was made with the TempliPhi DNA sequencing Template Amplification Kit (Amersham Biosciences). Sequencing was carried out on MegaBACE sequencing system using M13 forward primer and DYEnamic ET Dye Terminator Cycle Sequencing Kit for MegaBACE DNA analysis systems (Amersham Biosciences).

### Sequence analyses

Base-calling and vector screening were performed on the 4,977 obtained chromatograms using Phred, version 0.020425 [53]. The EST sequences were clustered using the Phrap assembler, version 0.990329 with the default settings [53], which yielded 478 preliminary contigs with two or more sequences. 990 sequences did not show any significant overlap (singlets). Both contigs and singlets were screened for high quality sequences using the pruneEST script (obtained from Daniel Nilsson, Karolinska Institutet, Sweden) which trims the sequences based on the quality scores. The minimal length of high quality sequence was set to 100 bp. Two contigs and 194 singlets did not match the set criteria and were removed from further analyses. A single contig were identified to be a chimera and were split into two contigs. 18S ribosomal RNA (rRNA) sequences were identified in two singlets, and one contig was identified to contain a 28S rRNA sequence; these were removed from further analyses. Sequences with inserts in the opposite direction were identified as starting with a stretch of Ts (8), or as having significant matches in the wrong direction to known genes (3); these were reversed. This procedure resulted in 476 contigs with two reads or more and 794 singlets. The average and median lengths of these 1,270 sequences were 537 and 482 bp, respectively.

A stretch of eight or more As were in the end of the EST is indicative of mRNA polyadenylation; 179 such ESTs were identified. Based on sequence similarity to genes in the databases 134 of these were annotated as coding genes (see below). The regions around the 3' end of these genes were aligned based on the position of the termination codons (UGA), and a sequence logo was created using WebLogo [54].

The sequence data were deposited in dbEST at NCBI with the accession numbers GW585169-GW589878.

### Databases and similarity searches

All databases used in the analyses were downloaded in May 2008. The non-redundant protein database were downloaded from the National Center for Biotechnology Information (NCBI) [45]. To broaden the taxonomic sampling of microbial eukaryotes the protein databases from the genome projects of the oomyctete *Phytophthora ramorum *and *Phytophthora sojae*, the diatoms *Thalassiosira pseudonana *and *Phaeodactylum tricornutum*, the green alga *Chlamydomonas reinhardtii*, and the heterolobosean *Naegleria gruberi *were downloaded from the US Department of Energy Joint Genome Institute [55]. Protein translations of 168,875 EST clusters from 54 diverse eukaryotic lineages from Taxonomically Broad EST Database (TBestDB) [56] were also included. Similarity searches using BLASTX, version 2.2.17 [57] were performed for the 1,270 unique sequences against the downloaded protein databases. 831 sequences gave a hit with an E value less than 1e^-5^. Putative open reading frames (orfs) were extracted using the indication of the coding frame from the BLAST result file and translated into putative protein sequences.

### Phylogenetic analyses

Phylogenetic analyses were performed on a putative selenophosphate synthetase (SelD) homolog. An automatic phylogenetic tree was generated using the Phylogenie package [58], as described previously [14]. 28 sequences from the obtained tree were selected to represent the diversity of SelD in the three domains of life. 160 unambiguously aligned amino acid positions were identified by eye. Using Modelgenerator [59], BLOSUM62 + Γ was identified as the optimal substitution model. Maximum likelihood analysis with 500 bootstrap replicates was performed with RAxML, version 7.0.4 [60]. Bayesian analyses with two independent runs for 500,000 generations were performed with MrBayes, version 3.1.2 [61], using the default settings, except for the optimal substitution models. The first 100,000 generations were discarded as burnin.

### Codon usage analyses

The 831 sequences, for which putative orfs could be assigned, corresponded to 682 unique groups using the criteria of clustering sequences with pairwise BLASTN matches of <1e^-20^. The frequencies of G+C in fourfold degenerate positions (GC3_s_) and (N_c_'), a measure of the effective number of codons used in a gene which take the background nucleotide composition into account [41], were calculated using INCA [62]. A correspondence analysis on the relative synonymous codon usage (RSCU) values using the software CodonW [63] was performed to examine the variation of codon usage among genes.

245 of these 682 groups were found to have BLASTP matches to putative proteins from *S. salmonicida *[14] with E values <1e^-40 ^spanning 100 or more aligned amino acid positions. In twelve cases two different *S. barkhanus *orfs matched the same *S. salmonicida *orf; only the pair with the lowest E value was retained for further analyses. The amino acid and nucleotide sequences for the aligned homologous regions of the 233 putative orthologous pairs of *S. barkhanus *and *S. salmonicida *sequences were extracted. GC3_s _and N_c_' were calculated using INCA [62] for the orthologs. For each orthologous pair the amino acid sequences were aligned using ClustalW [64], and this alignment was used as a guide to align the nucleotide sequences using the transAlign.pl software [65]. Synonymous (d_s_) and nonsynonymous (d_N_) substitution rates were calculating using the Yang and Nielsen method [66] using the PAML program package [67].

### Allelic sequence variation

Polybayes.pl is a software designed to identify single nucleotide polymorphism (SNP) within assemblies of sequences produced using the Sanger technology that takes quality values into account [42]. This tool was used to quantify the sequence heterogeneity within assembled clusters. The genes encoding enolase, ribosomal protein S2, glutamate dehydrogenase, heat shock protein 70 and pyruvate kinase were selected for further analyses of the observed intragenomic sequence heterogeneity. Verification and discovery of polymorphisms in fragments of the enolase, ribosomal protein S2, glutamate dehydrogenase, cytoplasmic heat shock protein 70 and pyruvate kinase in *S. barkhanus *and *S. salmonicida *were accomplished by cloning and sequencing of individual PCR clones. Low degeneracy primer-pairs targeting ~500 bp regions in both species were designed based on alignments of the genes (Additional file 9) and recommendations in the Phusion HotStart polymerase instruction manual. PCR reactions were performed in 1× Phusion HF buffer with 1.5 mM MgCl_2_, 200 μM dNTPs, 0.5 μM of forward and reverse primers, 40 ng *S. barkhanus *or *S. salmonicida *genomic DNA and 0.8 U Phusion HS DNA polymerase (Finnzymes) in a total volume of 40 μl. The reactions were incubated for 2 min at 98°C followed by 32 cycles of 98°C for 15 sec, 55°C for 30 sec, 72°C for 25 sec, and were then held at 4°C.

The PCR products were purified using the QIAquick PCR purification kit (Qiagen) and eluted in 30 μl ddH_2_O. Purified PCR products were A-tailed by adding 25 μl of respective eluate to one PuReTaq Ready-To-Go PCR bead (GE Healthcare) followed by incubation at 72°C for 10 min. A-tailed PCR fragments were cloned by the TOPO TA cloning Kit for Sequencing (Invitrogen) following the manufacturers' recommendations, except that the cloning reactions were desalted by dialyzing 1 hr on a 0.025 μm VSWP membrane filter (Millipore) against a large excess of ddH_2_O before transformation into TOP10 Electrocomp *E. coli *cells (Invitrogen).

For each gene, 10-14 clones were picked and grown over-night followed by plasmid mini-preps using NucleoSpin Plasmid kit (Macherey-Nagel). The plasmids were sequenced using -40 M13 forward primer at the Uppsala Genome Center using BigDye^® ^Terminator v3.1 chemistry and capillary electrophoresis on an ABI3730XL (Applied Biosystems). Two *S. barkhanus *genes were selected for deeper coverage. An additional ~50 clones of each of these were inoculated into 100 μl 2×YT-Kanamycin (50 μg/ml) in a multi-well culture plate and grown 20 hrs at 37°C without shaking. 1 μl of the culture was used for amplification with TempliPhi Amplification Kit (GE Healthcare) according to the manufacturers' recommendations. Sequencing reactions was performed as above. Post-reaction cleanup was performed with Sephadex G-50 (GE Healthcare) micro columns prepared in Multiscreen plates (Millipore).

### Genome size estimations

Genome sizes were estimated using PFGE and flow cytometry. For PFGE, exponentially growing cells were harvested by centrifugation (10 min at 2,500 × g, 4°C) and washed twice in cold TSE buffer (10 mM TrisHCl pH 8.0, 100 mM NaCl and 50 mM EDTA). The cells were resuspended in TSE buffer to the concentration of 2 - 10 × 10^8 ^cells/ml, and were equilibrated to 40°C. The cell suspension were mixed with an equal volume of 1.6% (w/v) InCert agarose gel (Lonza Rockland, Inc., Rockland, ME, USA) equilibrated to 42°C and solidified as plugs at 4°C. After 20 min, plugs containing approximately 1 × 10^7 ^cells/100 μl plug, were incubated in cell lysis buffer (1% Lauroyl Sarcosine Sodium Salt (Sigma), 0.5 M EDTA pH 8.0 and 2 mg/ml Proteinase K (Roche)) at 42°C for 48 h, changing the buffer after 24 h. The plugs were rinsed twice in TE buffer for 30 min and stored in TE buffer at 4°C.

Plugs containing chromosomal DNA were washed twice for 30 min in 0.5 × TBE buffer (80 mM Tris, 80 mM boric acid and 2 mM EDTA, pH 8.0) and directly loaded and sealed into wells of a 1% (w/v) Seakem GTG agarose gel (PFGE grade, Bio-Rad Laboratories, CA, USA). PFGE was performed in 0.5 × TBE using CHEF Mapper system (Bio-Rad). Different sets of running conditions were used for separation of the chromosomes. Chromosomes of *Saccharomyces cerevisiae *(0.225 - 2.2 Mb), *Hansenula wingei *(1.05 - 3.13 Mb) and *Schizosaccharomyces pombe *(3.5 - 5.7 Mb) (Bio-Rad) were used as standard DNA size markers. After electrophoresis, gels were stained with ethidium bromide (0.5 μg/ml) for 20 min, destained in distilled water for 30 min and photographed under UV-light. Densitometry analysis of the obtained bands was performed using the SynGene software.

Protists cells were fixed and analyzed for flow cytometry according to Bernander et al. [18].

## Authors' contributions

KR-L cultivated the organisms, designed and performed most of the molecular biology experiments, and drafted these parts of the manuscript. JJ-H performed PCR studies and drafted this part of the manuscript. AJ established *S. barkhanus *cultures. KT helped with the flow cytometry analyses. JOA and SGS initiated and supervised the study. SGS drafted part of the manuscript. JOA performed the bioinformatic studies, drafted most of manuscript, and coordinated the study. All authors read and approved the final manuscript.

## Supplementary Material

Additional file 1 ***S. barkhanus *morphology**. A movie recorded using a 40× magnification lens showing a *S. barkhanus *cell. Click here for file

Additional file 2 ***S. salmonicida *morphology**. A movie recorded using a 40× magnification lens showing a *S. salmonicida *cell. Click here for file

Additional file 3 **Swimming *S. barkhanus***. A movie recorded using a 20× magnification lens showing swimming *S. barkhanus *cells. Click here for file

Additional file 4 **Swimming *S. salmonicida***. A movie recorded using a 20× magnification lens showing swimming *S. salmonicida *cells. Click here for file

Additional file 5 **Clustered *S. barkhanus *ESTs with significant sequence similarities**. A table listing general properties of all *S. barkhanus *ESTs with significant sequence similarity to proteins in the public databases. Click here for file

Additional file 6 **Clustered ESTs without homologs in the *G. intestinalis *genome**. A table listing all *S. barkhanus *ESTs with significant sequence similarity to proteins in the public databases, but without homologs in *G. intestinalis*. The E values and taxonomic designation are given for the most similar sequences. Click here for file

Additional file 7 ***S. barkhanus *pyruvate kinase alleles**. An alignment of individual alleles of *S. barkhanus *pyruvate kinase identified in the PCR experiments. Click here for file

Additional file 8 **PFGE analyses of *S. barkhanus *and *S. salmonicida *chromosomal DNA**. The results of the PFGE experiments together with densitometry analyses and a discussion of the results. Click here for file

Additional file 9 **PCR primers for sequence heterogeneity studies**. A table of PCR primers used to study sequence heterogeneity in the *Spironucleus *enolase, ribosomal protein S2, glutamate dehydrogenase, hsp70, and pyruvate kinase genes. Click here for file

## References

[B1] McGrath CL Katz LA Genome diversity in microbial eukaryotes Trends Ecol Evol 2004 19 32 38 10.1016/j.tree.2003.10.007 16701223

[B2] Schurko AM Neiman M Logsdon JM Jr Signs of sex: what we know and how we know it Trends Ecol Evol 2009 24 208 217 10.1016/j.tree.2008.11.010 19282047

[B3] Adl SM Simpson AG Farmer MA Andersen RA Anderson OR Barta JR Bowser SS Brugerolle G Fensome RA Fredericq S The new higher level classification of eukaryotes with emphasis on the taxonomy of protists J Eukaryot Microbiol 2005 52 399 451 10.1111/j.1550-7408.2005.00053.x 16248873

[B4] Kolisko M Cepicka I Hampl V Leigh J Roger AJ Kulda J Simpson AG Flegr J Molecular phylogeny of diplomonads and enteromonads based on SSU rRNA, alpha-tubulin and HSP90 genes: implications for the evolutionary history of the double karyomastigont of diplomonads BMC Evol Biol 2008 8 205 10.1186/1471-2148-8-205 18627633 PMC2496913

[B5] Brugerolle G Lee JJ Lee JJ, Leedale GF, Bradbury P Order Diplomonadida An Illustrated Guide to the Protozoa 2002 2 Lawrence, Kansas: Society of Protozoologists 1125 1135

[B6] Monis PT Caccio SM Thompson RC Variation in *Giardia *: towards a taxonomic revision of the genus Trends Parasitol 2009 25 93 100 10.1016/j.pt.2008.11.006 19135417

[B7] Franzén O Jerlström-Hultqvist J Castro E Sherwood E Ankarklev J Reiner D Palm D Andersson JO Andersson B Svärd S Draft genome sequencing of *Giardia intestinalis *assemblage B isolate GS: are human giardiasis caused by two different species? PLoS Pathog 2009 5 8 10.1371/journal.ppat.1000560 PMC2723961 19696920

[B8] Kolisko M Cepicka I Hampl V Kulda J Flegr J The phylogenetic position of enteromonads: a challenge for the present models of diplomonad evolution Int J Syst Evol Microbiol 2005 55 1729 1733 10.1099/ijs.0.63542-0 16014510

[B9] Jørgensen A Sterud E Phylogeny of *Spironucleus *(Eopharyngia: Diplomonadida: Hexamitinae) Protist 2007 158 247 254 10.1016/j.protis.2006.12.003 17292667

[B10] Sterud E Poppe TT Bornø G Intracellular infection with *Spironucleus barkhanus *(Diplomonadida, Hexamitidae) in farmed Arctic char *Salvelinus alpinus* Dis Aquat Organ 2003 56 155 161 10.3354/dao056155 14598991

[B11] Jørgensen A Sterud E The marine pathogenic genotype of *Spironucleus barkhanus *from farmed salmonids redescribed as *Spironucleus salmonicida *n. sp J Eukaryot Microbiol 2006 53 531 541 10.1111/j.1550-7408.2006.00144.x 17123418

[B12] Kent ML Ellis J Fournie JW Dawe SC Bagshaw JW Whitaker DJ Systemic hexamitid (Protozoa, Diplomonadida) infection in seawater pen-reared Chinook salmon *Oncorhynchus *tshawytscha Dis Aquat Organ 1992 14 81 89 10.3354/dao014081

[B13] Poppe TT Mo TA Iversen L Disseminated hexamitosis in sea-caged Atlantic salmon *Salmo salar* Dis Aquat Organ 1992 14 91 97 10.3354/dao014091

[B14] Andersson JO Sjögren ÅM Horner DS Murphy CA Dyal PL Svärd SG Logsdon JM Jr Ragan MA Hirt RP Roger AJ A genomic survey of the fish parasite *Spironucleus salmonicida *indicates genomic plasticity among diplomonads and significant lateral gene transfer in eukaryote genome evolution BMC Genomics 2007 8 51 10.1186/1471-2164-8-51 17298675 PMC1805757

[B15] Joint Genome Institute - *Spironucleus vortens *genome project http://genome.jgi-psf.org/Spivo0/Spivo0.info.html

[B16] Morrison HG McArthur AG Gillin FD Aley SB Adam RD Olsen GJ Best AA Cande WZ Chen F Cipriano MJ Genomic minimalism in the early diverging intestinal parasite *Giardia lamblia* Science 2007 317 1921 1926 10.1126/science.1143837 17901334

[B17] Keeling PJ Doolittle WF Widespread and ancient distribution of a noncanonical genetic code in diplomonads Mol Biol Evol 1997 14 895 901 9287422 10.1093/oxfordjournals.molbev.a025832

[B18] Bernander R Palm JE Svärd SG Genome ploidy in different stages of the *Giardia lamblia *life cycle Cell Microbiol 2001 3 55 62 10.1046/j.1462-5822.2001.00094.x 11207620

[B19] Yu LZ Birky JCW Adam RD The two nuclei of *Giardia *each have complete copies of the genome and are partitioned equationally at cytokinesis Eukaryot Cell 2002 1 191 199 10.1128/EC.1.2.191-199.2002 12455954 PMC118032

[B20] Mark Welch D Meselson M Evidence for the evolution of bdelloid rotifers without sexual reproduction or genetic exchange Science 2000 288 1211 1215 10.1126/science.288.5469.1211 10817991

[B21] Morrison HG McArthur AG Gillin FD Aley SB Adam RD Olsen GJ Best AA Cande WZ Chen F Cipriano MJ Genomic minimalism in the early diverging intestinal parasite *Giardia lamblia* Science 2007 317 1921 1926 10.1126/science.1143837 17901334

[B22] Poxleitner MK Carpenter ML Mancuso JJ Wang CJ Dawson SC Cande WZ Evidence for karyogamy and exchange of genetic material in the binucleate intestinal parasite *Giardia intestinalis* Science 2008 319 1530 1533 10.1126/science.1153752 18339940

[B23] Adam RD Biology of *Giardia lamblia* Clin Microbiol Rev 2001 14 447 475 10.1128/CMR.14.3.447-475.2001 11432808 PMC88984

[B24] Biagini GA Yarlett N Ball GE Billetz AC Lindmark DG Martinez MP Lloyd D Edwards MR Bacterial-like energy metabolism in the amitochondriate protozoon *Hexamita inflata* Mol Biochem Parasitol 2003 128 11 19 10.1016/S0166-6851(03)00025-2 12706792

[B25] Andersson JO Hirt RP Foster PG Roger AJ Evolution of four gene families with patchy phylogenetic distribution: influx of genes into protist genomes BMC Evol Biol 2006 6 27 10.1186/1471-2148-6-27 16551352 PMC1484493

[B26] Nash TE Surface antigenic variation in *Giardia lamblia* Mol Microbiol 2002 45 585 590 10.1046/j.1365-2958.2002.03029.x 12139606

[B27] Weiland ME McArthur AG Morrison HG Sogin ML Svärd SG Annexin-like alpha giardins: a new cytoskeletal gene family in *Giardia lamblia* Int J Parasitol 2005 35 617 626 10.1016/j.ijpara.2004.12.009 15862575

[B28] Field J Rosenthal B Samuelson J Early lateral transfer of genes encoding malic enzyme, acetyl-CoA synthetase and alcohol dehydrogenases from anaerobic prokaryotes to *Entamoeba histolytica* Mol Microbiol 2000 38 446 455 10.1046/j.1365-2958.2000.02143.x 11069669

[B29] Andersson JO Sjögren ÅM Davis LAM Embley TM Roger AJ Phylogenetic analyses of diplomonad genes reveal frequent lateral gene transfers affecting eukaryotes Curr Biol 2003 13 94 104 10.1016/S0960-9822(03)00003-4 12546782

[B30] Andersson JO Gene transfer and diversification of microbial eukaryotes Annu Rev Microbiol 2009 63 177 193 10.1146/annurev.micro.091208.073203 19575565

[B31] Romero H Zhang Y Gladyshev VN Salinas G Evolution of selenium utilization traits Genome Biol 2005 6 R66 10.1186/gb-2005-6-8-r66 16086848 PMC1273633

[B32] Erpin project http://tagc.univ-mrs.fr/erpin/

[B33] Gautheret D Lambert A Direct RNA motif definition and identification from multiple sequence alignments using secondary structure profiles J Mol Biol 2001 313 1003 1011 10.1006/jmbi.2001.5102 11700055

[B34] Zhang Y Romero H Salinas G Gladyshev VN Dynamic evolution of selenocysteine utilization in bacteria: a balance between selenoprotein loss and evolution of selenocysteine from redox active cysteine residues Genome Biol 2006 7 17 10.1186/gb-2006-7-2-r17 PMC1794560 17054778

[B35] Carlton JM Hirt RP Silva JC Delcher AL Schatz M Zhao Q Wortman JR Bidwell SL Alsmark UC Besteiro S Draft genome sequence of the sexually transmitted pathogen *Trichomonas vaginalis* Science 2007 315 207 212 10.1126/science.1132894 17218520 PMC2080659

[B36] Que X Svärd SG Meng TC Hetsko ML Aley SB Gillin FD Developmentally regulated transcripts and evidence of differential mRNA processing in *Giardia lamblia* Mol Biochem Parasitol 1996 81 101 110 10.1016/0166-6851(96)02698-9 8892309

[B37] Mazumder B Seshadri V Fox PL Translational control by the 3'-UTR: the ends specify the means Trends Biochem Sci 2003 28 91 98 10.1016/S0968-0004(03)00002-1 12575997

[B38] Chen SL Lee W Hottes AK Shapiro L McAdams HH Codon usage between genomes is constrained by genome-wide mutational processes Proc Natl Acad Sci USA 2004 101 3480 3485 10.1073/pnas.0307827100 14990797 PMC373487

[B39] Knight RD Freeland SJ Landweber LF A simple model based on mutation and selection explains trends in codon and amino-acid usage and GC composition within and across genomes Genome Biol 2001 2 0010 10.1186/gb-2001-2-4-research0010 PMC31479 11305938

[B40] Lafay B Sharp PM Synonymous codon usage variation among *Giardia lamblia *genes and isolates Mol Biol Evol 1999 16 1484 1495 10555279 10.1093/oxfordjournals.molbev.a026060

[B41] Novembre JA Accounting for background nucleotide composition when measuring codon usage bias Mol Biol Evol 2002 19 1390 1394 12140252 10.1093/oxfordjournals.molbev.a004201

[B42] Marth GT Korf I Yandell MD Yeh RT Gu Z Zakeri H Stitziel NO Hillier L Kwok PY Gish WR A general approach to single-nucleotide polymorphism discovery Nat Genet 1999 23 452 456 10.1038/70570 10581034

[B43] Qiu XY Wu LY Huang HS McDonel PE Palumbo AV Tiedje JM Zhou JZ Evaluation of PCR-generated chimeras: mutations, and heteroduplexes with 16S rRNA gene-based cloning Appl Environ Microbiol 2001 67 880 887 10.1128/AEM.67.2.880-887.2001 11157258 PMC92662

[B44] Dawson SC Pham JK House SA Slawson EE Cronembold D Cande WZ Stable transformation of an episomal protein-tagging shuttle vector in the piscine diplomonad *Spironucleus vortens* BMC Microbiol 2008 8 71 10.1186/1471-2180-8-71 18445284 PMC2386477

[B45] National Center for Biotechnology Information http://www.ncbi.nlm.nih.gov/

[B46] Caccio SM Ryan U Molecular epidemiology of giardiasis Mol Biochem Parasitol 2008 160 75 80 10.1016/j.molbiopara.2008.04.006 18501440

[B47] Lebbad M Ankarklev J Tellez A Leiva B Andersson JO Svärd S Dominance of *Giardia *assemblage B in Leon, Nicaragua Acta Trop 2008 106 44 53 10.1016/j.actatropica.2008.01.004 18325480

[B48] Fan JB Korman SH Cantor CR Smith CL *Giardia lamblia *: haploid genome size determined by pulsed field gel electrophoresis is less than 12 Mb Nucleic Acids Res 1991 19 1905 1908 10.1093/nar/19.8.1905 2030969 PMC328122

[B49] Ramesh MA Malik SB Logsdon JM Jr A phylogenomic inventory of meiotic genes; evidence for sex in *Giardia *and an early eukaryotic origin of meiosis Curr Biol 2005 15 185 191 15668177 10.1016/j.cub.2005.01.003

[B50] Cooper MA Adam RD Worobey M Sterling CR Population genetics provides evidence for recombination in *Giardia* Curr Biol 2007 17 1984 1988 10.1016/j.cub.2007.10.020 17980591

[B51] Keister DB Axenic culture of *Giardia lamblia *in TYI-S-33 medium supplemented with bile Trans R Soc Trop Med Hyg 1983 77 487 488 10.1016/0035-9203(83)90120-7 6636276

[B52] Jørgensen A Sterud E SSU rRNA gene sequence reveals two genotypes of *Spironucleus barkhanus *(Diplomonadida) from farmed and wild Arctic charr *Salvelinus alpinus* Dis Aquat Organ 2004 62 93 96 10.3354/dao062093 15648835

[B53] Laboratory of Phil Green http://www.phrap.org/

[B54] Crooks GE Hon G Chandonia JM Brenner SE WebLogo: a sequence logo generator Genome Res 2004 14 1188 1190 10.1101/gr.849004 15173120 PMC419797

[B55] DOE Joint Genome Institute http://www.jgi.doe.gov/

[B56] O'Brien EA Koski LB Zhang Y Yang L Wang E Gray MW Burger G Lang BF TBestDB: a taxonomically broad database of expressed sequence tags (ESTs) Nucleic Acids Res 2007 35 D445 451 10.1093/nar/gkl770 17202165 PMC1899108

[B57] Altschul SF Madden TL Schaffer AA Zhang J Zhang Z Miller W Lipman DJ Gapped BLAST and PSI-BLAST: a new generation of protein database search programs Nucleic Acids Res 1997 25 3389 3402 10.1093/nar/25.17.3389 9254694 PMC146917

[B58] Frickey T Lupas AN PhyloGenie: automated phylome generation and analysis Nucleic Acids Res 2004 32 5231 5238 10.1093/nar/gkh867 15459293 PMC521674

[B59] ModelGenerator: amino acid and nucleotide substitution model selection http://bioinf.nuim.ie/software/modelgenerator

[B60] Stamatakis A RAxML-VI-HPC: maximum likelihood-based phylogenetic analyses with thousands of taxa and mixed models Bioinformatics 2006 22 2688 2690 10.1093/bioinformatics/btl446 16928733

[B61] Huelsenbeck JP Ronquist F MRBAYES: Bayesian inference of phylogenetic trees Bioinformatics 2001 17 754 755 10.1093/bioinformatics/17.8.754 11524383

[B62] Supek F Vlahovicek K INCA: synonymous codon usage analysis and clustering by means of self-organizing map Bioinformatics 2004 20 2329 2330 10.1093/bioinformatics/bth238 15059815

[B63] Correspondence analysis of codon usage http://codonw.sourceforge.net/

[B64] Thompson JD Gibson TJ Plewniak F Jeanmougin F Higgins DG The CLUSTAL_X windows interface: flexible strategies for multiple sequence alignment aided by quality analysis tools Nucleic Acids Res 1997 25 4876 4882 10.1093/nar/25.24.4876 9396791 PMC147148

[B65] Bininda-Emonds OR transAlign: using amino acids to facilitate the multiple alignment of protein-coding DNA sequences BMC Bioinformatics 2005 6 156 10.1186/1471-2105-6-156 PMC1175081 15969769

[B66] Yang Z Nielsen R Estimating synonymous and nonsynonymous substitution rates under realistic evolutionary models Mol Biol Evol 2000 17 32 43 10666704 10.1093/oxfordjournals.molbev.a026236

[B67] Yang Z PAML 4: phylogenetic analysis by maximum likelihood Mol Biol Evol 2007 24 1586 1591 10.1093/molbev/msm088 17483113

