# Supplementary material for: Large genomic differences between the morphologically indistinguishable diplomonads Spironucleus barkhanus and Spironucleus salmonicida
Source: BMC Genomics. 2010 Apr 21;11:258. doi: 10.1186/1471-2164-11-258 (PMC2874811; doi:10.1186/1471-2164-11-258)
Supplement: Additional file 5 — Clustered S. barkhanus ESTs with significant sequence similarities. A table listing general properties of all S. barkhanus ESTs with significant sequence similarity to proteins in the public databases. [file 1471-2164-11-258-S5.PDF]

Additional file 5 - Roxström-Lindquist, *et al.*

Properties of clustered *S. barkhanus* ESTs with significant sequence similarities.

Contig	*Annotation*	*Class[a]*	*#ESTs*	*Freqency of cysteine*	*#SNPs*	*Group*	*#Contigs in group*
Contig414	1,4-alpha-glucan branching enzyme	GB	4	1,2%			
Contig554	14-3-3 protein	GB	21	1,2%	1		
Contig530	2,3-bisphosphoglycerate-independent phosphoglycerate mutase	GNB	15	1,1%	9	Contig530	3
Contig476	2,3-bisphosphoglycerate-independent phosphoglycerate mutase	GNB	8	1,2%	3	Contig530	3
Contig307	2,3-bisphosphoglycerate-independent phosphoglycerate mutase	GNB	3	1,4%	1	Contig530	3
Contig291	20S proteasome alpha subunit 1	GB	3	2,9%			
SBBE-F69	20S proteasome alpha subunit 2	GNB	1	1,2%			
Contig297	20S proteasome alpha subunit 3	GNB	3	0,0%			
SBAI-F88	20S proteasome alpha subunit 4	GB	1	0,7%			
Contig279	20S proteasome alpha subunit 6	GNB	2	0,0%			
Contig392	26S protease regulatory subunit 6A	GNB	4	1,2%	2		
SBBB-F68	26S protease regulatory subunit 6B	GB	1	0,6%			
SBBQ-F4	26S protease regulatory subunit 7	GNB	1	0,6%			
SBAJ-F60	26S protease regulatory subunit 7	GNB	1	2,1%			
SBAT-F48	26S protease regulatory subunit 7	GNB	1	1,3%			
Contig278	26S protease regulatory subunit 8	GNB	2	2,3%		Contig278	2
Contig245	26S protease regulatory subunit 8	GNB	2	3,4%	4	Contig278	2
Contig62	26S protease regulatory subunit 8	GNB	1	3,1%			
Contig200	26S proteasome ATPase subunit S4, putative	GB	2	1,0%		Contig200	3
Contig92	26S proteasome ATPase subunit S4, putative	GB	1	1,2%		Contig200	3
Contig30	26S proteasome ATPase subunit S4, putative	GB	1	0,6%		Contig200	3
SBBC-F43	26S proteasome ATPase subunit S4, putative	GB	1	1,4%			
SBBJ-F59	26S proteasome non-ATPase regulatory subunit 2	GB	1	0,7%			
SBBM-F4	26S proteasome non-ATPase regulatory subunit 7	GNB	1	2,2%			
Contig393	40S ribosomal protein S19	SU	4	2,3%			
SBBB-F71	4-alpha-glucanotransferase, amylo-alpha-1,6-glucosidase	GNB	1	3,4%			
Contig312	4-methyl-5-thiazole monophosphate biosynthesis enzyme	GNB	3	4,8%			
Contig68	5' nucleotidase family protein	GNB	1	2,0%		Contig68	2
Contig12	5' nucleotidase family protein	GB	1	1,2%		Contig68	2
Contig314	70 kDa peptidylprolyl isomerase, putative	GB	3	1,6%			
Contig49	AAA family ATPase	GNB	1	0,0%		Contig156	2
Contig156	AAA family ATPase	GB	2	1,1%		Contig156	2
SBAZ-F7	AAA family ATPase	GB	1	1,2%			
Contig76	ABC transporter	GB	1	2,6%		Contig76	2
Contig80	ABC transporter	GNB	1	2,2%		Contig76	2
Contig470	ABC transporter family protein	GB	7	1,8%			
SBAD-F64	ABC transporter family protein	GB	1	3,7%			
SBBC-F73	ABC transporter family protein	GB	1	2,5%			
SBBD-F2	ABC transporter family protein	GB	1	3,2%			
SBBP-F59	ABC transporter, ATP-binding protein	GB	1	3,7%			
SBBZ-F66	ABC transporter, ATP-binding protein	GB	1	1,5%			
Contig375	ABC transporter, ATP-binding protein, putative	GB	3	0,9%			
Contig535	Acetyl-CoA synthetase	GB	15	2,0%	1	Contig535	2
Contig211	Acetyl-CoA synthetase	GB	2	3,1%		Contig535	2

Contig569	Acidic ribosomal protein P0	GB	29	1,2%	2		
SBAP-F19	Acidic ribosomal protein P0	GB	1	2,5%			
Contig560	Actin related protein	GB	23	2,2%	1	Contig560	3
Contig537	Actin related protein	GB	16	2,6%	2	Contig560	3
Contig64	Actin related protein	GB	1	1,8%		Contig560	3
Contig343	Actophorin	SU	3	1,5%			
SBBC-F67	Acyl-CoA synthetase	GB	1	1,4%			
SBBT-F90	Adenylate kinase	GB	1	0,8%			
Contig369	ADP-ribosylation factor	GB	3	0,0%			
Contig137	Alanine aminotransferase, putative	GB	2	3,8%		Contig423	2
Contig423	Alanine aminotransferase, putative	GB	5	3,4%	3	Contig423	2
Contig341	Alanine aminotransferase, putative	GB	3	1,4%			
Contig228	Alanine aminotransferase, putative	GB	2	1,7%			
SBAP-F12	Alanyl-tRNA synthetase	GB	1	5,7%			
Contig575	Alcohol dehydrogenase	GB	38	3,5%	6		
SBAT-F32	Alcohol dehydrogenase	GNB	1	2,6%			
Contig333	Alcohol dehydrogenase	GB	3	2,4%			
Contig153	aldose 1-epimerase	SU	2	1,2%	2		
Contig376	Aldose reductase	GNB	3	1,8%	1		
Contig396	Alpha adaptin	GB	4	4,6%	4		
Contig389	Alpha-1 giardin	GNB	4	2,0%		Contig389	2
Contig397	Alpha-1 giardin	GNB	4	1,3%			
Contig118	Alpha-1 giardin	GNB	2	0,0%			
Contig459	Alpha-14 giardin	GNB	6	3,0%	1	Contig459	4
Contig24	Alpha-14 giardin	GNB	1	3,2%		Contig459	4
Contig160	Alpha-5 giardin	GNB	2	0,5%		Contig160	2
Contig109	Alpha-5 giardin	GNB	1	0,0%		Contig160	2
Contig350	Alpha-5 giardin	GNB	3	1,7%		Contig459	4
Contig430	Alpha-5 giardin	GNB	5	1,8%	2	Contig459	4
Contig434	Alpha-5 giardin	GNB	5	1,2%	1		
SBBG-F31	Alpha-SNAP, putative	GB	1	1,3%			
Contig148	Alpha-tubulin	GNB	2	3,6%		Contig566	7
Contig566	Alpha-tubulin	GB	28	3,1%	3	Contig566	7
Contig465	Alpha-tubulin	GB	7	3,5%	1	Contig566	7
Contig332	Alpha-tubulin	GB	3	4,2%		Contig566	7
Contig316	Alpha-tubulin	GB	3	3,5%	2	Contig566	7
Contig8	Alpha-tubulin	GB	1	2,9%		Contig566	7
Contig319	Alpha-tubulin	GB	3	2,9%	1	Contig566	7
SBBP-F86	Amino acid transporter family	GB	1	3,0%			
SBBL-F35	Amino acid transporter family	GB	1	1,3%			
Contig224	Amino acid transporter system N2, putative	GB	2	2,8%			
Contig532	Aminoacyl-histidine dipeptidase	GB	15	6,0%		Contig532	2
Contig521	Aminoacyl-histidine dipeptidase	GB	14	6,6%	4	Contig532	2
SBAO-F83	Aminoacyl-histidine dipeptidase	GNB	1	1,8%			
SBBI-F50	Aminoacyl-histidine dipeptidase	GB	1	3,3%			
Contig382	Aminopeptidase I	SU	3	1,4%	2		
SBAZ-F44	Aminotransferase	SU	1	3,2%			
SBBY-F7	ARF GAP	GNB	1	1,2%			
SBBU-F95	Arginyl-tRNA synthetase	GNB	1	0,0%			
SBAU-F17	Arginyl-tRNA synthetase	GB	1	0,6%			
Contig210	ARL1	GB	2	3,2%			
Contig178	Arsenical pump-driving ATPase	GNB	2	0,0%		Contig178	2
Contig84	Arsenical pump-driving ATPase	GNB	1	0,0%		Contig178	2
Contig198	Aspartate aminotransferase, cytoplasmic	GB	2	1,3%			
Contig145	aspartyl aminopeptidase	SU	2	3,2%			
Contig411	Aspartyl-tRNA synthetase	GNB	4	0,4%	3		
Contig269	Aspartyl-tRNA synthetase	GB	2	1,4%			
Contig305	ATP/GTP binding protein, putative	GNB	3	0,5%			
Contig199	ATP-binding cassette protein 5	GB	2	1,7%			
SBAM-F13	ATP-dependent DNA helicase recQ	GNB	1	0,6%			

Contig83	ATP-dependent RNA helicase	SU	1	1,9%		Contig125	3
Contig262	ATP-dependent RNA helicase p54, putative	GB	2	1,1%			
Contig229	ATP-dependent RNA helicase-like protein	GNB	2	0,6%			
SBBN-F68	ATP-dependent RNA helicase-like protein	GB	1	2,2%			
Contig512	A-type flavoprotein	GB	12	2,0%	4	Contig512	2
Contig326	A-type flavoprotein	GB	3	1,6%		Contig512	2
SBAS-F52	A-type flavoprotein	GB	1	2,5%			
Contig409	Axonemal p66.0	GB	4	0,7%			
Contig415	Axoneme central apparatus protein	GB	4	2,0%		Contig415	2
Contig308	Axoneme central apparatus protein	GB	3	1,2%	1	Contig415	2
Contig265	Beta adaptin	GNB	2	1,6%			
SBBF-F31	Beta adaptin	GB	1	1,4%			
Contig584	Beta tubulin	GNB	54	2,6%	5		
Contig542	Bip	GB	18	0,2%	3		
Contig418	Branched-chain amino acid aminotransferase	GB	4	2,6%	1		
SBAN-F45	Calmodulin	GNB	1	1,8%			
SBBQ-F21	Calmodulin	GB	1	0,0%			
Contig128	Caltractin	GB	2	0,0%			
Contig141	CAMP-dependent protein kinase regulatory chain	GB	2	0,6%			
Contig464	Carbamate kinase	GB	7	3,3%	5		
Contig177	carotenoid isomerase	SU	2	2,2%			
Contig97	carotenoid isomerase	SU	1	1,5%			
SBBZ-F32	Cathepsin B precursor	GB	1	4,3%			
SBAR-F44	Cathepsin B precursor	GB	1	4,7%			
Contig161	Cathepsin B precursor	GB	2	5,5%			
SBAH-F2	Cathepsin B precursor	GB	1	5,3%			
SBBU-F38	Cathepsin L precursor	GB	1	1,9%			
SBAC-F20	CDC50	GB	1	1,9%			
Contig345	Centromere/microtubule binding protein CBF5	GB	3	1,9%		Contig345	2
Contig56	Centromere/microtubule binding protein CBF5	GB	1	3,6%		Contig345	2
Contig501	Chaperone protein DnaJ	GB	10	4,2%	1	Contig507	2
Contig507	Chaperone protein DnaJ	GB	11	4,6%		Contig507	2
Contig539	Chaperone protein DnaJ	GB	16	1,7%	2	Contig539	2
Contig516	Chaperone protein DnaJ	GB	13	2,3%		Contig539	2
SBAH-F62	Chaperone protein dnaJ	GB	1	2,0%			
SBBM-F83	Chaperonin 60	GNB	1	2,0%			
Contig441	Clathrin heavy chain	GB	5	1,2%			
Contig417	Clathrin heavy chain	GB	4	2,5%	1		
SBAU-F52	Clathrin heavy chain	GB	1	1,3%			
Contig586	ClpB protein	GB	60	1,4%	6	Contig586	2
Contig567	ClpB protein	GB	28	0,8%		Contig586	2
SBAT-F31	Clusterin associated protein 1, putative	GB	1	0,0%			
Contig293	Coatomer alpha subunit	GNB	3	1,9%		Contig293	2
SBBA-F76	Coiled-coil protein	GB	1	0,6%			
Contig272	Coiled-coil protein	GB	2	0,0%			
SBAM-F87	Coiled-coil protein	GB	1	0,6%			
SBBX-F66	Coiled-coil protein	GB	1	3,7%			
SBCA-F2	Coiled-coil protein	GB	1	0,0%			
Contig28	Conserved hypothertical protein	SU	1	3,2%		Contig293	2
Contig220	Conserved hypothertical protein	SU	2	1,9%		Contig389	2
SBAJ-F74	Conserved hypothertical protein	SU	1	1,3%			
Contig395	Conserved hypothertical protein	SU	4	1,7%	3		
Contig347	Conserved hypothertical protein	SU	3	3,3%			
SBAY-F56	Conserved hypothertical protein	SU	1	0,6%			
SBBU-F89	Conserved hypothertical protein	SU	1	2,4%			
Contig241	Conserved hypothertical protein	SU	2	0,0%			

Contig163	Conserved hypothertical protein	SU	2	0,6%	1		
Contig344	Conserved hypothertical protein	SU	3	0,4%			
SBAD-F94	Conserved hypothertical protein	SU	1	2,9%			
SBBU-F84	Conserved hypothertical protein	SU	1	2,6%			
SBBK-F47	Conserved hypothertical protein	SU	1	3,3%			
SBAU-F80	Conserved hypothertical protein	SU	1	1,2%			
SBBY-F20	Conserved hypothertical protein	SU	1	0,0%			
SBBL-F84	Conserved hypothertical protein	SU	1	0,0%			
SBAQ-F87	Conserved hypothertical protein	SU	1	2,5%			
SBBL-F11	Conserved hypothertical protein	SU	1	0,6%			
SBAR-F54	Conserved hypothertical protein	SU	1	0,6%			
SBAZ-F12	Conserved hypothertical protein	SU	1	0,0%			
SBBH-F14	Conserved hypothertical protein	SU	1	0,6%			
SBBV-F69	Conserved hypothertical protein	SU	1	0,6%			
SBBG-F68	Conserved hypothertical protein	SU	1	0,0%			
Contig149	Conserved hypothertical protein	SU	2	2,4%			
Contig136	Conserved hypothertical protein	SU	2	4,4%			
SBBY-F39	Conserved hypothertical protein	SU	1	0,6%			
SBBN-F35	Conserved hypothertical protein	SU	1	0,0%			
Contig235	Conserved hypothertical protein	SU	2	0,7%			
Contig130	Conserved hypothertical protein	SU	2	0,6%			
SBAX-F16	Conserved hypothertical protein	SU	1	0,0%			
Contig216	Conserved hypothertical protein	SU	2	0,6%			
Contig361	Conserved hypothertical protein	SU	3	0,6%			
SBAR-F88	Copine I	GB	1	1,3%			
Contig243	CTP synthase	GNB	2	0,7%			
SBBB-F27	CTP synthase	GNB	1	0,7%			
SBAD-F24	CTP synthase/UTP-ammonia lyase	GNB	1	3,8%			
Contig324	CXC-rich protein	GNB	3	16,0%	1	Contig324	2
Contig391	CXC-rich protein	GNB	4	18,1%		Contig391	5
Contig432	CXC-rich protein	GNB	5	17,3%		Contig526	5
Contig9	CXC-rich protein	GNB	1	17,7%		Contig60	2
Contig99	CXC-rich protein	GNB	1	15,9%		Contig99	2
Contig358	CXC-rich protein	GNB	3	15,4%			
Contig295	Cysteine desulfurase	GB	3	1,8%			
SBBX-F18	Cysteinyl-tRNA synthetase	GB	1	0,7%			
Contig591	Cytosolic HSP70	GB	451	0,8%	9	Contig591	2
Contig549	Cytosolic HSP70	GB	20	0,5%	1	Contig591	2
SBBV-F27	DEAD box RNA helicase Vasa	GNB	1	2,7%			
Contig78	Deoxyhypusine synthase, putative	GB	1	2,3%		Contig266	2
Contig266	Deoxyhypusine synthase, putative	GB	2	1,3%		Contig266	2
SBBZ-F81	Deoxyhypusine synthase, putative	GB	1	5,0%			
SBAV-F67	Deoxynucleoside kinase	GB	1	1,3%			
SBBV-F72	Deoxyribose-phosphate aldolase	GB	1	6,1%			
Contig483	Desulfoferrodoxin	SU	8	2,0%	2		
SBAH-F36	Developmentally regulated GTP-binding protein 1	GB	1	0,0%			
Contig165	DinF protein	GB	2	1,2%			
SBAS-F68	DinF protein	GB	1	0,0%			
Contig281	Dipeptidyl-peptidase III	GB	2	0,8%			
SBBO-F40	Diphthine synthase	GB	1	1,3%			
SBBX-F33	DNA-dependent ATPase, putative	GB	1	1,3%			
SBBQ-F57	DNA-directed RNA polymerase II largest subunit RPB1	GB	1	1,9%			
Contig169	DNA-directed RNA polymerase RPB2	GB	2	3,4%			
SBAD-F30	DNA-directed RNA polymerase RPB2	GB	1	3,4%			
SBBN-F58	DNA-directed RNA polymerase subunit H	SU	1	1,9%			
SBBU-F5	dTDP-D-glucose 4,6-dehydratase	SU	1	2,9%			
Contig552	Dynamin	GB	20	0,9%	1		

Contig342	Dynein binding protein, putative	GB	3	2,4%	2	Contig342	2
Contig94	Dynein binding protein, putative	GB	1	1,6%		Contig342	2
SBAL-F80	Dynein heavy chain	GNB	1	1,8%			
SBAF-F7	Dynein heavy chain	GNB	1	1,2%			
Contig270	Dynein heavy chain	GNB	2	1,8%	2		
SBAK-F8	Dynein heavy chain	GNB	1	1,3%			
SBAO-F44	Dynein heavy chain	GNB	1	1,8%			
SBAC-F31	Dynein heavy chain	GB	1	2,0%			
Contig357	Dynein heavy chain	GB	3	2,9%			
SBAG-F3	Dynein heavy chain	GB	1	0,6%			
SBAX-F10	Dynein heavy chain	GB	1	0,6%			
SBBY-F65	Dynein heavy chain	GB	1	2,3%			
SBAC-F77	Dynein heavy chain	GB	1	3,2%			
SBAM-F2	Dynein heavy chain	GB	1	0,6%			
SBAT-F82	Dynein heavy chain	GB	1	1,3%			
SBBR-F68	Dynein heavy chain	GB	1	1,4%			
SBBT-F80	Dynein heavy chain, putative	GB	1	1,4%			
SBBH-F84	Dynein intermediate chain	GB	1	1,3%			
SBBP-F69	Dynein intermediate chain	GB	1	0,0%			
SBBS-F21	Dynein intermediate chain	GB	1	0,6%			
SBBV-F24	Dynein light chain	GNB	1	2,2%			
SBAX-F5	Dynein light chain	GB	1	0,0%			
Contig370	Dynein light intermediate chain	GB	3	1,5%			
SBAI-F93	Dynein regulatory complex	GB	1	0,0%			
Contig315	Dynein-like protein	GB	3	1,0%			
Contig247	E04F6.2 like protein	GB	2	0,7%			
Contig254	EH domain binding protein epsin 2	GB	2	0,5%			
Contig587	Elongation factor 1-alpha	GB	67	2,0%	4		
Contig410	Elongation factor 1-gamma	GB	4	3,2%		Contig460	2
Contig460	Elongation factor 1-gamma	GB	6	2,5%		Contig460	2
Contig581	Elongation factor 2	GB	45	2,5%	16		
SBBH-F53	Endothelin-converting enzyme 2	GB	1	1,5%			
Contig583	Enolase	GB	47	3,2%	6		
SBAC-F53	ER lumen protein retaining receptor	GB	1	1,9%			
Contig176	Eukaryotic peptide chain release factor subunit 1	GB	2	3,0%		Contig176	2
Contig47	Eukaryotic peptide chain release factor subunit 1	GB	1	3,8%		Contig176	2
Contig492	Eukaryotic translation initiation factor 5A	GB	9	2,7%	3		
SBAL-F82	Fatty acid elongase 1	GB	1	1,9%			
Contig42	Fe-hydrogenase-1	GNB	1	5,3%		Contig294	2
Contig294	Fe-hydrogenase-1	GNB	3	4,5%		Contig294	2
Contig217	Fe-hydrogenase-1	GNB	2	4,4%			
SBBF-F6	Fe-hydrogenase-1	GNB	1	2,6%			
SBAU-F67	Fe-hydrogenase-1	GNB	1	1,9%			
Contig135	Fe-hydrogenase-1	GB	2	6,8%			
SBBM-F82	Fibrillarin-like pre-rRNA processing protein Narcisi et al	GB	1	0,0%			
Contig400	FixW protein, putative	GB	4	3,1%		Contig466	2
Contig466	FixW protein, putative	GB	7	3,1%		Contig466	2
SBAB-F53	FKBP-type peptidyl-prolyl cis-trans isomerase	GNB	1	1,5%			
SBAO-F94	FKBP-type peptidyl-prolyl cis-trans isomerase	GNB	1	0,0%			
Contig172	Flagella associated protein	GB	2	2,4%			
SBBN-F75	Flagella associated protein	GB	1	4,9%			
Contig439	Fructokinase	SU	5	1,2%			
Contig529	Fructose-bisphosphate aldolase	GB	15	1,5%	1	Contig578	2
Contig578	Fructose-bisphosphate aldolase	GB	40	1,6%	4	Contig578	2
Contig493	Giardia trophozoite antigen GTA-2	GB	9	2,3%	2		
Contig288	Glucokinase	GNB	2	2,9%		Contig288	2

Contig257	Glucokinase	GNB	2	3,0%		Contig288	2
SBBB-F43	Glucokinase	GNB	1	3,0%			
Contig456	Glucose-6-phosphate isomerase	GNB	6	1,4%		Contig456	3
Contig322	Glucose-6-phosphate isomerase	GNB	3	0,6%		Contig456	3
Contig277	Glucose-6-phosphate isomerase	GNB	2	0,6%	1	Contig456	3
Contig284	Glutamate synthase	GNB	2	1,4%			
SBBK-F28	Glutamate synthase	GB	1	6,9%			
Contig227	Glutaminyl-tRNA synthetase	GB	2	1,3%			
SBAI-F91	Glutaminyl-tRNA synthetase	GB	1	2,7%			
SBCA-F17	Glutaminyl-tRNA synthetase	GB	1	0,7%			
Contig576	Glyceraldehyde 3-phosphate dehydrogenase	GB	39	3,3%	3	Contig576	2
Contig300	Glyceraldehyde 3-phosphate dehydrogenase	GB	3	2,6%		Contig576	2
SBBD-F95	Glycerol kinase	GNB	1	4,4%			
Contig388	Glycogen phosphorylase	GB	4	2,1%		Contig458	2
Contig458	Glycogen phosphorylase	GB	6	2,0%		Contig458	2
Contig349	Glycogen phosphorylase	GB	3	1,2%			
SBBU-F75	Glycogen synthase, putative	GB	1	1,5%			
SBBD-F22	Glycogen synthase, putative	GB	1	1,3%			
Contig334	Glycyl-tRNA synthetase	GB	3	1,8%	3		
Contig401	GTP-binding nuclear protein RAN/TC4	GB	4	1,5%			
Contig179	GTP-binding protein, putative	GB	2	2,6%			
Contig304	Heat shock protein 70	GB	3	0,6%	3		
Contig590	Heat shock protein HSP 90-alpha	GNB	83	1,3%	1	Contig515	3
Contig555	Heat-shock protein, putative	GB	22	1,2%	7	Contig555	3
Contig468	Heat-shock protein, putative	GB	7	0,8%		Contig555	3
Contig174	Heat-shock protein, putative	GB	2	3,0%		Contig555	3
Contig134	Hexose transporter	GNB	2	1,2%		Contig473	2
Contig473	Hexose transporter	GB	8	1,3%	1	Contig473	2
Contig39	High cysteine membrane protein Group 1	GNB	1	17,5%		Contig100	5
SBBJ-F29	High cysteine membrane protein Group 1	GNB	1	8,3%			
Contig121	High cysteine membrane protein Group 2	GNB	2	14,9%		Contig100	5
Contig16	High cysteine membrane protein Group 2	GNB	1	14,1%		Contig100	5
Contig11	High cysteine membrane protein Group 2	GNB	1	16,0%		Contig324	2
Contig162	High cysteine membrane protein Group 2	GNB	2	18,2%		Contig351	2
Contig351	High cysteine membrane protein Group 2	GNB	3	17,9%		Contig351	2
Contig110	High cysteine membrane protein Group 2	GNB	1	15,0%		Contig372	2
Contig538	High cysteine membrane protein Group 2	GNB	16	17,3%		Contig538	5
Contig479	High cysteine membrane protein Group 2	GNB	8	17,3%		Contig538	5
Contig203	High cysteine membrane protein Group 2	GNB	2	18,0%		Contig538	5
Contig53	High cysteine membrane protein Group 2	GNB	1	18,4%		Contig538	5
Contig7	High cysteine membrane protein Group 2	GNB	1	17,9%		Contig538	5
Contig159	High cysteine membrane protein Group 2	GNB	2	15,5%			
Contig259	High cysteine membrane protein Group 2	GNB	2	16,8%			
SBCB-F74	High cysteine membrane protein Group 2	GNB	1	16,6%			
Contig373	High cysteine membrane protein Group 2	GNB	3	18,4%			
SBBG-F23	High cysteine membrane protein Group 2	GNB	1	17,7%			
SBBJ-F23	High cysteine membrane protein Group 2	GNB	1	17,2%			
Contig25	High cysteine membrane protein Group 2	GNB	1	17,6%			
Contig158	High cysteine membrane protein Group 2	GNB	2	18,5%			
SBAK-F55	High cysteine membrane protein Group 2	GNB	1	16,2%			
SBBR-F81	High cysteine membrane protein Group 2	GNB	1	16,6%			
Contig367	High cysteine membrane protein Group 5	GNB	3	17,3%		Contig100	5
Contig301	High cysteine membrane protein Group 5	GNB	3	17,3%		Contig100	5
Contig368	High cysteine membrane protein Group 5	GNB	3	18,0%		Contig391	5
Contig366	High cysteine membrane protein Group 5	GNB	3	17,1%		Contig526	5
Contig138	High cysteine membrane protein Group 5	GNB	2	16,6%	2	Contig538	5
Contig219	High cysteine membrane protein Group 5	GNB	2	16,6%		Contig538	5
Contig60	High cysteine membrane protein Group 5	GNB	1	18,2%		Contig60	2
Contig21	High cysteine membrane protein Group 5	GNB	1	14,1%			
Contig32	High cysteine membrane protein TMK-like	GNB	1	17,0%		Contig526	5

SBCB-F5	High cysteine membrane protein TMK-like	GNB	1	15,7%			
SBBC-F3	High cysteine membrane protein TMK-like	GNB	1	18,3%			
SBBL-F49	High cysteine membrane protein TMK-like	GNB	1	17,0%			
SBBV-F32	High cysteine membrane protein TMK-like	GB	1	17,9%			
Contig526	High cysteine membrane protein VSP-like	GNB	14	17,0%	1	Contig526	5
SBCA-F42	High cysteine membrane protein VSP-like	GNB	1	15,5%			
SBBX-F41	High cysteine membrane protein VSP-like	GNB	1	17,3%			
Contig70	High cysteine protein	GB	1	15,2%		Contig324	2
SBBJ-F35	Histidyl-tRNA synthetase	GB	1	1,4%			
Contig124	Histone deacetylase	GB	2	3,1%			
Contig189	Histone H2A	GB	2	0,0%			
Contig22	Histone H3	GB	1	0,7%		Contig292	3
Contig29	Histone H3	GB	1	0,7%		Contig292	3
Contig292	Histone H3	GB	3	0,7%		Contig292	3
Contig403	Histone H3	GB	4	0,7%		Contig403	5
Contig263	Histone H3	GB	2	0,7%		Contig403	5
Contig81	Histone H3	GB	1	0,7%		Contig403	5
Contig107	Histone H3	GB	1	0,8%		Contig403	5
Contig6	Histone H3	GB	1	0,0%		Contig403	5
SBAZ-F8	Histone H3 methyltransferase complex, subunit CPS60/ASH2/BRE2	SU	1	1,3%			
Contig313	Histone H4	GB	3	0,0%			
SBAS-F30	HpaII tiny fragments locus 9c	GNB	1	2,5%			
SBAO-F79	H-SHIPPO 1	GNB	1	0,6%			
SBBP-F37	H-SHIPPO 1	GB	1	0,0%			
Contig338	H-SHIPPO 1	GB	3	0,7%	2		
Contig282	H-SHIPPO 1	GB	2	1,3%	2		
Contig171	Hypothetical protein	GB	2	0,7%	1	Contig171	2
Contig79	Hypothetical protein	GNB	1	3,4%		Contig230	2
Contig230	Hypothetical protein	GNB	2	3,4%		Contig230	2
Contig240	Hypothetical protein	GNB	2	0,7%		Contig240	2
Contig500	Hypothetical protein	GNB	10	1,4%	2	Contig419	3
Contig36	Hypothetical protein	GNB	1	0,8%		Contig61	3
Contig61	Hypothetical protein	GNB	1	0,7%		Contig61	3
Contig67	Hypothetical protein	GB	1	0,8%		Contig67	2
Contig356	Hypothetical protein	GNB	3	1,1%			
SBAJ-F23	Hypothetical protein	GNB	1	0,0%			
Contig195	Hypothetical protein	GNB	2	1,2%			
Contig310	Hypothetical protein	GNB	3	2,9%			
SBAV-F58	Hypothetical protein	GNB	1	2,8%			
SBAU-F51	Hypothetical protein	GNB	1	0,6%			
SBCB-F33	Hypothetical protein	GNB	1	4,1%			
Contig152	Hypothetical protein	GNB	2	1,5%			
Contig302	Hypothetical protein	GNB	3	0,9%			
Contig374	Hypothetical protein	GNB	3	2,9%			
SBAT-F86	Hypothetical protein	GNB	1	1,3%			
Contig126	Hypothetical protein	GB	2	12,4%			
SBCB-F61	Hypothetical protein	GB	1	2,1%			
Contig147	Hypothetical protein	GB	2	0,0%			
Contig558	Hypothetical protein	GB	22	3,1%	2		
SBAT-F59	Hypothetical protein	GB	1	1,9%			
Contig303	Hypothetical protein	GB	3	0,6%			
SBAM-F85	Hypothetical protein	GB	1	1,8%			
Contig231	Hypothetical protein	GB	2	2,4%	1		
Contig446	Hypothetical protein	GB	6	1,8%	3		
SBAS-F57	Hypothetical protein	GB	1	0,6%			
SBBX-F5	Hypothetical protein	GB	1	0,6%			
Contig273	Hypothetical protein	GB	2	1,5%			
SBAH-F25	Hypothetical protein	GB	1	0,0%			
SBBE-F93	Hypothetical protein	GB	1	1,8%			

SBBJ-F25	Hypothetical protein	GB	1	1,3%	
SBAN-F2	Hypothetical protein	GB	1	2,5%	
SBBP-F18	Hypothetical protein	GB	1	0,0%	
Contig206	Hypothetical protein	GB	2	1,3%	
SBBZ-F77	Hypothetical protein	GB	1	1,5%	
SBBV-F49	Hypothetical protein	GB	1	1,1%	
SBBZ-F76	Hypothetical protein	GB	1	1,4%	
SBAK-F79	Hypothetical protein	GB	1	1,1%	
SBBS-F82	Hypothetical protein	GB	1	1,9%	
Contig363	Hypothetical protein	GB	3	1,5%	
SBBX-F54	Hypothetical protein	GB	1	2,2%	
SBAI-F82	Hypothetical protein	GB	1	0,0%	
SBBL-F38	Hypothetical protein	GB	1	0,7%	
SBBQ-F7	Hypothetical protein	GB	1	0,0%	
SBAO-F74	Hypothetical protein	GB	1	0,6%	
Contig274	Hypothetical protein	GB	2	4,3%	
SBAF-F11	Hypothetical protein	GB	1	0,0%	
SBAM-F43	Hypothetical protein	GB	1	1,0%	
Contig212	Hypothetical protein	GB	2	0,6%	
Contig214	Hypothetical protein	GB	2	2,6%	
Contig215	Hypothetical protein	GB	2	1,3%	
Contig234	Hypothetical protein	GB	2	1,3%	
Contig246	Hypothetical protein	GB	2	2,5%	
Contig264	Hypothetical protein	GB	2	1,8%	
Contig267	Hypothetical protein	GB	2	0,6%	
Contig321	Hypothetical protein	GB	3	0,8%	
Contig331	Hypothetical protein	GB	3	1,1%	2
SBAG-F43	Hypothetical protein	GB	1	1,3%	
SBAH-F37	Hypothetical protein	GB	1	0,0%	
SBAH-F88	Hypothetical protein	GB	1	5,7%	
SBAK-F52	Hypothetical protein	GB	1	0,7%	
SBAM-F94	Hypothetical protein	GB	1	0,0%	
SBAN-F76	Hypothetical protein	GB	1	1,8%	
SBAP-F68	Hypothetical protein	GB	1	0,6%	
SBAQ-F8	Hypothetical protein	GB	1	0,8%	
SBAS-F39	Hypothetical protein	GB	1	3,8%	
SBAV-F80	Hypothetical protein	GB	1	0,7%	
SBAX-F12	Hypothetical protein	GB	1	0,6%	
SBAY-F55	Hypothetical protein	GB	1	0,6%	
SBBA-F58	Hypothetical protein	GB	1	0,6%	
SBBC-F74	Hypothetical protein	GB	1	0,0%	
SBBD-F42	Hypothetical protein	GB	1	1,9%	
SBBD-F43	Hypothetical protein	GB	1	1,3%	
SBBD-F57	Hypothetical protein	GB	1	0,0%	
SBBE-F74	Hypothetical protein	GB	1	1,7%	
SBBH-F55	Hypothetical protein	GB	1	2,0%	
SBBO-F21	Hypothetical protein	GB	1	1,8%	
SBBP-F96	Hypothetical protein	GB	1	0,6%	
SBBV-F40	Hypothetical protein	GB	1	0,7%	
SBBX-F35	Hypothetical protein	GB	1	0,6%	
SBBY-F28	Hypothetical protein	GB	1	1,5%	
SBBY-F40	Hypothetical protein	GB	1	3,5%	
SBBZ-F39	Hypothetical protein	GB	1	2,0%	
SBCB-F68	Hypothetical protein	GB	1	0,0%	
Contig276	HZGJ	GB	2	1,2%	
SBAV-F50	IFT complex B	GB	1	0,0%	
SBAI-F47	IFT complex B	GB	1	0,0%	
SBBH-F36	Importin beta-3 subunit	GB	1	1,8%	
Contig151	Intraflagellar transport protein component IFT74/72	GB	2	0,4%	

Contig202	Isoleucyl-tRNA synthetase	GNB	2	2,9%	1		
SBAA-F46	Kinase	GB	1	1,4%			
Contig197	Kinase, AGC PKA	GB	2	0,0%			
Contig239	Kinase, CAMK CAMKL	GNB	2	3,9%			
SBAT-F54	Kinase, CAMK CAMKL	GNB	1	2,6%			
SBAR-F25	Kinase, CAMK CAMKL	GNB	1	0,6%			
Contig154	Kinase, CAMK CAMKL	GB	2	2,3%			
SBBF-F46	Kinase, CAMK CAMKL	GB	1	3,2%			
SBAF-F21	Kinase, CAMK CAMKL	GB	1	0,6%			
SBAV-F48	Kinase, CDC7	GB	1	0,0%			
Contig360	Kinase, CK1 Casein kinase	GB	3	1,2%	1		
SBBA-F59	Kinase, CMGC CDK	GB	1	0,0%			
Contig205	Kinase, CMGC MAPK	GNB	2	1,2%			
SBAT-F43	Kinase, CMGC MAPK	GNB	1	0,0%			
Contig402	Kinase, CMGC MAPK	GB	4	1,8%			
SBAN-F17	Kinase, CMGC MAPK	GB	1	0,8%			
SBBI-F74	Kinase, NEK	GB	1	1,3%			
SBCB-F58	Kinase, NEK	GB	1	1,3%			
Contig248	Kinase, NEK	GB	2	1,8%			
Contig252	Kinase, NEK	GB	2	1,2%			
SBAZ-F75	Kinase, PLK	GB	1	1,9%			
Contig144	Kinase, putative	GB	2	2,5%			
Contig190	Kinase, STE STE20	GB	2	1,8%			
SBCB-F36	Kinase, STE STE20	GB	1	2,3%			
SBBS-F63	Kinesin-16	GB	1	1,3%			
SBBB-F15	Kinesin-5	GB	1	0,0%			
SBAC-F44	Kinesin-9	GB	1	1,2%			
Contig131	La ribonucleoprotein, putative	GB	2	0,0%			
Contig271	Leucyl-tRNA synthetase	GB	2	2,7%			
SBBQ-F39	Long chain fatty acid CoA ligase 5	GB	1	1,1%			
SBBD-F78	Long chain fatty acid CoA ligase, putative	GB	1	2,4%			
SBBR-F13	Long chain fatty acid CoA ligase, putative	GB	1	1,2%			
Contig115	Lysosomal acid phosphatase precursor	GNB	2	1,4%			
SBBG-F24	Macrophage migration inhibitory factor	GNB	1	0,9%			
SBAJ-F88	Malate dehydrogenase	GNB	1	3,9%			
Contig540	Malate dehydrogenase	GB	16	3,4%			
Contig551	Malic enzyme	GB	20	3,1%	3		
SBAG-F4	Manganese-dependent inorganic pyrophosphatase, putative	GB	1	1,2%			
SBAZ-F66	Manganese-dependent inorganic pyrophosphatase, putative	GB	1	2,3%			
SBBC-F26	MCM5	GB	1	4,7%			
SBCB-F25	MCM7	GB	1	0,6%			
SBBD-F15	MCT-1 protein-like protein	GB	1	1,3%			
Contig196	MDR-type permease	GB	2	2,5%			
Contig261	MDR-type permease	GB	2	3,9%			
SBAX-F63	Metalloprotease, insulinase family	GNB	1	0,6%			
Contig318	Methionine aminopeptidase	GB	3	1,7%	3		
SBAJ-F43	Methionyl-tRNA synthetase	GB	1	4,0%			
Contig117	Methionyl-tRNA synthetase	GB	2	3,3%			
SBBI-F34	Molybdenum cofactor sulfurase	GB	1	1,2%			
Contig487	Mu adaptin	GB	9	1,6%	3		
Contig127	Multidrug resistance protein B	GNB	2	0,6%		Contig127	2
Contig287	Multidrug resistance-associated protein 1	GB	2	0,0%		Contig488	2
Contig503	Multidrug resistance-associated protein 1	GB	11	2,4%			
SBBE-F32	Multidrug resistance-associated protein 1	GB	1	0,0%			
SBAB-F90	Myb 1-like protein	GB	1	1,3%			
SBBC-F38	Myb 1-like protein	GB	1	3,5%			
SBAR-F3	Na+ driven multidrug efflux pump	GB	1	1,3%			
SBAN-F63	Na+ driven multidrug efflux pump	GB	1	1,3%			

SBBG-F44	N-acetyltransferase-like protein	GNB	1	2,9%			
SBBR-F45	NADH oxidase	GB	1	1,3%			
Contig559	NADH oxidase	GNB	23	2,3%	1		
SBCA-F48	NADPH oxidoreductase, putative	GNB	1	1,5%			
Contig390	NADP-specific glutamate dehydrogenase	GB	4	1,1%	2		
Contig462	NADP-specific glutamate dehydrogenase	GB	7	1,2%	1		
SBBP-F61	Neurogenic locus Notch protein precursor	GNB	1	11,9%			
Contig193	Neurogenic locus Notch protein precursor	GB	2	14,2%			
Contig325	Neurogenic locus Notch protein precursor	GB	3	14,8%			
SBAN-F30	Nif3-related protein	GB	1	2,5%			
Contig255	Nitroreductase	SU	2	2,7%			
SBAV-F7	Nitroreductase family protein fused to ferredoxin domain Fd-NR1	GNB	1	20,4%			
SBBG-F21	Nitroreductase Fd-NR2	GNB	1	7,1%			
SBAC-F29	NOD3 protein, putative	GB	1	3,0%			
SBBL-F28	Nonsense-mediated mRNA decay protein 3	GB	1	4,7%			
SBBU-F45	Nuclear ATP/GTP-binding protein	GB	1	2,4%			
Contig285	Nucleolar GAR1-like protein, putative	GNB	2	0,5%			
SBBP-F4	Nucleolar GTPase	GB	1	0,0%			
SBAX-F80	Nucleolar GTP-binding protein 1, putative	GB	1	0,6%			
Contig113	Nucleolar protein NOP2	GB	1	0,0%		Contig113	3
Contig31	Nucleolar protein NOP2	GB	1	0,0%		Contig113	3
Contig51	Nucleolar protein NOP2	GB	1	0,0%		Contig113	3
SBAP-F37	Nucleolar protein NOP5	GNB	1	1,3%			
Contig298	Nucleoside diphosphate kinase	GB	3	2,0%	1		
SBBB-F84	Nucleoside diphosphate kinase	GB	1	1,3%			
Contig413	Nucleotide-binding protein 1	GB	4	3,9%		Contig449	3
Contig449	Nucleotide-binding protein 1	GB	6	4,7%	4	Contig449	3
Contig37	Nucleotide-binding protein 1	GB	1	3,1%		Contig449	3
Contig233	Ornithine carbamoyltransferase	GB	2	3,7%		Contig233	3
Contig10	Ornithine carbamoyltransferase	GB	1	3,5%		Contig233	3
Contig90	Ornithine carbamoyltransferase	GB	1	2,6%		Contig233	3
SBAG-F80	Ornithine decarboxylase	GB	1	2,4%			
SBAD-F57	Ornithine decarboxylase	GB	1	1,9%			
SBAR-F18	O-sialoglycoprotein endopeptidase	GNB	1	2,0%			
Contig183	Palmitoyl-protein thioesterase 3	SU	2	2,0%			
SBAH-F81	Palmitoyl-protein thioesterase 3	SU	1	2,7%			
SBBD-F49	PcnA	GB	1	2,8%			
Contig157	Peptidase T	SU	2	3,1%			
SBBH-F70	Peptidase T	SU	1	1,7%			
Contig485	Peptidyl-prolyl cis-trans isomerase B precursor	GB	9	3,1%	1		
Contig296	Peroxiredoxin 1	GNB	3	4,8%		Contig556	5
Contig556	Peroxiredoxin 1	GB	22	4,1%	2	Contig556	5
Contig412	Peroxiredoxin 1	GB	4	4,7%	1	Contig556	5
Contig463	Peroxiredoxin 1	GB	7	5,0%	1	Contig556	5
Contig55	Peroxiredoxin 1	GB	1	5,1%		Contig556	5
Contig112	Phosphatase	GB	1	2,8%		Contig112	2
SBAR-F11	Phosphatase	GNB	1	5,4%			
Contig188	Phosphatase	GNB	2	2,5%			
SBBZ-F30	Phosphatase	GB	1	3,8%			
SBBI-F60	Phosphatase	GB	1	3,5%			
SBBN-F2	Phosphatidyl synthase	GNB	1	1,2%			
Contig133	Phosphatidylinositol-4-phosphate 5-kinase, putative	GB	2	0,6%			
Contig469	Phosphatidylinositol-4-phosphate 5-kinase, putative	GNB	7	1,5%			
SBAJ-F94	Phosphoacetylglucosamine mutase	GB	1	2,5%			
Contig429	Phosphoglycerate kinase	GB	5	1,9%		Contig527	2
Contig527	Phosphoglycerate kinase	GB	14	2,0%	2	Contig527	2

Contig225	Phosphomannomutase-2	GNB	2	4,2%		Contig520	5
Contig170	Phosphomannomutase-2	GNB	2	4,5%		Contig520	5
Contig328	Phosphomannomutase-2	GNB	3	2,4%		Contig520	5
Contig461	Phosphomannomutase-2	GB	7	3,1%	1	Contig520	5
Contig520	Phosphomannomutase-2	GB	14	3,0%		Contig520	5
Contig236	Phosphomannomutase-2	GB	2	2,9%		Contig520	5
SBBI-F40	Plasma membrane calcium-transporting ATPase 2	GB	1	7,2%			
SBBD-F17	Plasma membrane calcium-transporting ATPase 2	GB	1	1,5%			
Contig445	Polyadenylate-binding protein, putative	GB	6	2,2%		Contig445	2
Contig444	Polyadenylate-binding protein, putative	GB	5	1,8%		Contig445	2
Contig377	Potassium-transporting ATPase alpha chain 1	GNB	3	1,6%			
Contig146	Potassium-transporting ATPase alpha chain 1	GB	2	1,6%			
SBAA-F53	Prolyl-tRNA synthetase	GNB	1	2,5%			
Contig381	Proteasome subunit beta type 1	GB	3	1,1%	2		
SBBI-F58	Proteasome subunit beta type 2	GNB	1	1,3%			
Contig120	Proteasome subunit beta type 4 precursor	GB	2	2,1%			
Contig364	Proteasome subunit beta type 5 precursor	GB	3	0,8%			
Contig96	Proteasome subunit beta type 7 precursor	GNB	1	3,0%		Contig98	2
Contig98	Proteasome subunit beta type 7 precursor	GNB	1	3,2%		Contig98	2
SBAY-F9	Protein 21.1	GB	1	0,0%			
Contig168	Protein disulfide isomerase PDI1	GB	2	2,4%	1		
Contig385	Protein disulfide isomerase PDI2	GB	4	2,5%	2		
Contig582	Protein disulfide isomerase PDI4	GB	46	1,7%		Contig582	2
Contig5	Protein disulfide isomerase PDI4	GB	1	1,7%		Contig582	2
SBAX-F56	Protein F10G7.1	GB	1	1,3%			
SBCB-F50	Protein F54C1.5	GB	1	1,9%			
SBBL-F19	Protein LRP16	GB	1	1,2%			
SBBC-F90	Protein phosphatase 2A B' regulatory subunit Wdb1	GB	1	1,4%			
Contig123	Protein phosphatase PP2A regulatory subunit A	GB	2	2,6%			
SBAP-F86	Pseudouridylate synthase, putative	GNB	1	1,3%			
SBBP-F33	Putative glucokinase, ROK family protein	SU	1	0,6%			
Contig510	Pyrophosphate-fructose 6-phosphate 1-phosphotransferase alpha subunit	GNB	12	1,8%			
Contig533	Pyruvate kinase	GB	15	4,5%	4	Contig533	2
Contig352	Pyruvate kinase	GB	3	3,4%	1	Contig533	2
Contig438	Pyruvate-flavodoxin oxidoreductase	GNB	5	3,2%		Contig438	2
Contig238	Pyruvate-flavodoxin oxidoreductase	GNB	2	4,2%		Contig438	2
Contig187	Pyruvate-flavodoxin oxidoreductase	GNB	2	2,3%			
Contig182	Pyruvate-flavodoxin oxidoreductase	GB	2	2,3%			
SBBG-F74	Pyruvate-flavodoxin oxidoreductase	GB	1	1,3%			
SBAI-F51	Rab GDI	GNB	1	1,5%			
SBAO-F35	Rab11	GNB	1	2,1%			
SBAG-F35	Rab1a	GB	1	0,6%			
Contig268	Rac/Rho-like protein	GNB	2	2,2%			
SBAQ-F45	Radial-spoke protein	GB	1	0,7%			
Contig125	Repetitive protein	SU	2	1,3%		Contig125	3
Contig46	Repetitive protein	SU	1	1,9%		Contig125	3
SBAO-F29	Replication factor C, subunit 5	GB	1	1,9%			
SBBY-F46	Ribonuclease, putative	GB	1	2,5%			
Contig544	Ribosomal protein L10	GB	19	2,9%	4	Contig572	2
Contig553	Ribosomal protein L10a	GB	21	2,3%	2		
Contig518	Ribosomal protein L11	GB	13	1,2%	5		
Contig497	Ribosomal protein L12	GB	10	1,1%	2		
Contig477	Ribosomal protein L13	GB	8	2,1%	2	Contig547	3
Contig495	Ribosomal protein L13	GB	10	2,2%	2	Contig547	3

Contig547	Ribosomal protein L13	GB	19	2,2%	1	Contig547	3
Contig548	Ribosomal protein L13a	GNB	20	3,1%	3		
Contig570	Ribosomal protein L14	GNB	30	2,4%	2		
Contig562	Ribosomal protein L15	GB	24	2,5%		Contig562	2
Contig2	Ribosomal protein L15	GB	1	3,7%		Contig562	2
Contig523	Ribosomal protein L17	GB	14	2,4%	1		
Contig531	Ribosomal protein L18	GB	15	2,2%			
Contig475	Ribosomal protein L18a	GB	8	0,6%	4		
Contig573	Ribosomal protein L19	GB	37	0,6%	2		
Contig585	Ribosomal protein L2	GNB	58	2,8%	4		
Contig505	Ribosomal protein L21	GNB	11	1,3%			
Contig579	Ribosomal protein L23	GB	41	2,2%	3		
Contig536	Ribosomal protein L23A	GB	16	0,7%	4		
Contig482	Ribosomal protein L24A	GB	8	2,0%			
Contig511	Ribosomal protein L26	GB	12	0,8%	2		
Contig524	Ribosomal protein L27	GNB	14	0,0%	2		
Contig550	Ribosomal protein L27a	GB	20	2,8%	3		
Contig565	Ribosomal protein L3	GB	27	3,1%	2	Contig565	2
Contig447	Ribosomal protein L3	GB	6	3,8%		Contig565	2
Contig437	Ribosomal protein L30	GNB	5	0,0%			
Contig451	Ribosomal protein L31B	GNB	6	2,0%			
Contig506	Ribosomal protein L32	GB	11	2,3%		Contig506	2
Contig431	Ribosomal protein L32	GB	5	2,4%		Contig506	2
Contig504	Ribosomal protein L34	GNB	11	3,5%	1		
Contig478	Ribosomal protein L35	GNB	8	0,7%		Contig478	2
Contig420	Ribosomal protein L35	GNB	4	0,8%		Contig478	2
SBAT-F10	Ribosomal protein L35a	GNB	1	3,6%			
Contig384	Ribosomal protein L36-1	GB	4	1,2%			
Contig309	Ribosomal protein L37	GB	3	5,2%			
Contig491	Ribosomal protein L37a	GB	9	5,8%	1		
Contig515	Ribosomal protein L4	GB	12	2,5%	3	Contig515	3
Contig543	Ribosomal protein L4	GB	19	1,9%	4	Contig515	3
Contig577	Ribosomal protein L5	GB	39	1,8%		Contig577	2
Contig436	Ribosomal protein L5	GB	5	3,6%		Contig577	2
Contig545	Ribosomal protein L7	GB	19	0,5%	5		
Contig563	Ribosomal protein L7a	GB	26	0,9%	4		
SBAX-F18	Ribosomal protein L7Ae	GB	1	5,1%			
Contig467	Ribosomal protein L9	GB	7	1,7%	7	Contig592	4
Contig592	Ribosomal protein L9	GB	9	1,7%	5	Contig592	4
Contig517	Ribosomal protein L9	GB	13	1,7%	8	Contig592	4
Contig244	Ribosomal protein L9	GB	2	1,7%		Contig592	4
Contig435	Ribosomal protein P1B	GB	5	0,9%	1	Contig419	3
Contig419	Ribosomal protein P1B	GB	4	1,0%		Contig419	3
Contig365	Ribosomal protein S10B	GB	3	3,4%			
Contig509	Ribosomal protein S11	GB	12	2,7%	2		
Contig522	Ribosomal protein S12	GNB	14	2,4%	1		
Contig481	Ribosomal protein S13	GB	8	1,3%	1		
Contig557	Ribosomal protein S14	GB	22	0,0%	2	Contig557	2
Contig4	Ribosomal protein S14	GB	1	1,0%		Contig557	2
Contig499	Ribosomal protein S15	GNB	10	0,0%	1		
Contig440	Ribosomal protein S15A	GB	5	2,3%			
Contig484	Ribosomal protein S16	GB	8	0,7%	2	Contig484	2
Contig348	Ribosomal protein S16	GB	3	0,7%	1	Contig484	2
Contig486	Ribosomal protein S17	GB	9	0,8%	1		
Contig408	Ribosomal protein S18	GB	4	2,0%	2		
Contig580	Ribosomal protein S2	GB	44	0,9%			
Contig339	Ribosomal protein S20	GB	3	2,8%			
Contig399	Ribosomal protein S21	GNB	4	1,2%			
Contig546	Ribosomal protein S23	GB	19	2,9%	1	Contig546	2
Contig18	Ribosomal protein S23	GB	1	2,9%		Contig546	2

Contig472	Ribosomal protein S24	GB	8	2,4%		Contig472	3
Contig50	Ribosomal protein S24	GB	1	2,5%		Contig472	3
Contig33	Ribosomal protein S24	GB	1	3,2%		Contig472	3
Contig489	Ribosomal protein S26	GB	9	5,1%			
SBAT-F41	Ribosomal protein S27	GNB	1	8,2%			
Contig175	Ribosomal protein S28	GB	2	1,6%			
Contig424	Ribosomal protein S29A	GB	5	10,4%			
Contig498	Ribosomal protein S3	GB	10	2,2%	1		
Contig488	Ribosomal protein S3a	GB	9	2,1%	2	Contig488	2
Contig568	Ribosomal protein S4	GB	29	1,2%	2	Contig568	2
Contig26	Ribosomal protein S4	GB	1	1,4%		Contig568	2
Contig571	Ribosomal protein S5	GB	31	2,1%	4		
Contig428	Ribosomal protein S6	GB	5	2,2%		Contig574	2
Contig574	Ribosomal protein S6	GB	37	2,2%	2	Contig574	2
Contig593	Ribosomal protein S7	GNB	8	1,8%			
Contig519	Ribosomal protein S8	GB	14	1,8%	3		
Contig496	Ribosomal protein S9	GB	10	0,0%	1		
Contig508	Ribosomal protein SA	GB	11	2,2%	4		
SBAB-F77	RNA polymerase AI large subunit	GNB	1	0,0%			
SBAG-F47	RNA polymerase AI large subunit	GB	1	3,1%			
SBAL-F13	RNA polymerase II subunit Rpb5a	GB	1	1,8%			
SBAJ-F17	RNase L inhibitor	GB	1	5,5%			
Contig427	Rubrerythrin	SU	5	2,8%	1	Contig427	2
Contig58	Rubrerythrin	SU	1	1,2%		Contig427	2
Contig564	Rubrerythrin	SU	27	2,6%	1		
Contig13	S-adenosylmethionine synthetase	GNB	1	1,7%		Contig106	2
Contig106	S-adenosylmethionine synthetase	GNB	1	1,3%		Contig106	2
SBBM-F38	Sec61-alpha	GB	1	1,9%			
Contig142	Sec61-alpha	GB	2	1,2%			
SBBC-F56	Selenophosphate synthetase	SU	1	3,5%			
Contig253	Selenoprotein W1	GB	2	3,3%			
SBBF-F96	Ser/Thr phosphatase 2A, 65kDa reg sub A	GB	1	3,2%			
Contig140	Ser/Thr phosphatase PP2A-2 catalytic subunit	GB	2	2,4%			
SBCA-F49	Serine peptidase, putative	GB	1	2,5%			
SBBC-F33	Serine/Threonine protein phosphatase	GB	1	1,7%			
SBBD-F7	Serine/threonine protein phosphatase 5	GB	1	3,3%			
SBBL-F31	Serine/threonine protein phosphatase 5	GB	1	5,2%			
SBBD-F92	Serine/threonine protein phosphatase 5	GB	1	0,0%			
SBAO-F1	Serine/threonine protein phosphatase 5	GB	1	2,4%			
Contig119	Serine/threonine protein phosphatase 7	GB	2	1,2%			
Contig426	Serine/threonine protein phosphatase 7	GB	5	3,7%	4		
Contig232	Seryl-tRNA synthetase	GB	2	2,1%		Contig443	6
Contig383	Seryl-tRNA synthetase	GB	4	1,4%		Contig443	6
Contig359	Seryl-tRNA synthetase	GB	3	1,8%		Contig443	6
Contig443	Seryl-tRNA synthetase	GB	5	1,2%		Contig443	6
Contig75	Seryl-tRNA synthetase	GB	1	1,3%		Contig443	6
Contig54	Seryl-tRNA synthetase	GB	1	1,3%		Contig443	6
Contig114	Sgt1-like protein	GB	2	0,6%			
SBAV-F69	Sigma adaptin	GNB	1	2,9%			
Contig406	Small glutamine-rich tetratricopeptide repeat-containing protein	GB	4	0,6%			
SBAF-F89	Spindle pole protein, putative	GNB	1	0,0%			
SBAD-F78	Spindle pole protein, putative	GB	1	4,3%			
Contig534	Stress-induced-phosphoprotein 1	GB	15	1,7%	5	Contig534	2
Contig474	Stress-induced-phosphoprotein 1	GB	8	0,6%	1	Contig534	2
SBAH-F48	SUA5 protein	GB	1	2,6%			
Contig289	TBP-interacting protein TIP49	GB	2	0,6%			
SBBH-F95	TBP-interacting protein TIP49	GB	1	0,0%			
SBBH-F40	TBP-interacting protein TIP49	GB	1	0,0%			

SBBA-F95	T-complex protein-10	GNB	1	0,6%			
Contig299	TCP-1 chaperonin subunit alpha	GB	3	1,5%			
Contig320	TCP-1 chaperonin subunit alpha	GB	3	2,3%			
Contig386	TCP-1 chaperonin subunit beta	GB	4	2,0%			
Contig398	TCP-1 chaperonin subunit beta	GB	4	1,0%			
Contig103	TCP-1 chaperonin subunit epsilon	GB	1	1,2%		Contig104	2
Contig104	TCP-1 chaperonin subunit epsilon	GB	1	1,2%		Contig104	2
Contig91	TCP-1 chaperonin subunit gamma	GNB	1	1,2%		Contig422	4
Contig73	TCP-1 chaperonin subunit gamma	GNB	1	4,9%		Contig422	4
Contig422	TCP-1 chaperonin subunit gamma	GB	5	2,4%	1	Contig422	4
Contig41	TCP-1 chaperonin subunit gamma	GB	1	3,1%		Contig422	4
Contig38	TCP-1 chaperonin subunit theta	GB	1	1,9%		Contig63	2
Contig63	TCP-1 chaperonin subunit theta	GB	1	1,3%		Contig63	2
Contig455	TCP-1 chaperonin subunit theta	GB	6	0,6%			
Contig45	TCP-1 chaperonin subunit zeta	GNB	1	2,5%		Contig95	2
Contig95	TCP-1 chaperonin subunit zeta	GNB	1	2,6%		Contig95	2
Contig330	TCP-1 chaperonin subunit zeta	GNB	3	1,9%	1		
SBCB-F27	Tem-1-like protein	GB	1	2,2%			
Contig372	Tenascin precursor	GB	3	13,4%		Contig372	2
SBCB-F72	Tenascin precursor	GNB	1	13,2%			
Contig1	Tenascin-like	GNB	1	14,0%		Contig19	2
Contig19	Tenascin-like	GB	1	15,7%		Contig19	2
Contig450	Thioredoxin reductase	GB	6	2,4%	4	Contig490	2
Contig490	Thioredoxin reductase	GB	9	2,9%		Contig490	2
Contig327	Thioredoxin reductase	GB	3	3,4%			
SBBR-F60	Threonine dehydratase	GB	1	1,0%			
SBAV-F26	Threonyl-tRNA synthetase	GB	1	1,2%			
SBAC-F96	Tip elongation aberrant protein 1	GB	1	0,6%			
Contig362	Transketolase	GNB	3	3,5%	2		
Contig378	Transketolase	GB	3	1,7%			
Contig471	Translation elongation factor	GB	8	3,1%			
SBBA-F46	Translation initiation factor 6	GB	1	1,4%			
Contig514	Translation initiation factor eIF-4A, putative	GNB	12	2,0%			
SBAI-F1	Translation initiation factor IF-2, putative	GB	1	1,3%			
SBBC-F52	Translation initiation factor IF-2, putative	GB	1	1,5%			
Contig404	Translationally controlled tumor protein-like protein	GB	4	3,1%			
SBBS-F4	TRAPPC5/Trs31	GNB	1	2,8%			
SBAZ-F5	Trichohyalin	GB	1	0,0%			
SBAG-F38	Trichohyalin	GB	1	0,6%			
Contig525	Triosephosphate isomerase, cytosolic	GNB	14	1,1%	4		
SBBV-F66	tRNA 2-methylthioadenosine synthase	GB	1	3,0%			
Contig407	Tryptophanyl-tRNA synthetase	GB	4	1,8%			
SBAN-F41	Tubulin specific chaperone B	GB	1	0,6%			
SBBP-F36	Tubulin tyrosine ligase	GB	1	4,0%			
SBBU-F28	U2 small nuclear ribonucleoprotein A' putative	GB	1	2,3%			
Contig129	U2 small nuclear ribonucleoprotein A', putative	GNB	2	2,3%			
Contig494	Ubiquitin	GNB	10	3,2%		Contig502	3
Contig502	Ubiquitin	GNB	10	3,2%		Contig502	3
Contig354	Ubiquitin	GNB	3	3,3%		Contig502	3
SBAY-F83	Ubiquitin carboxyl-terminal hydrolase 14	GB	1	1,2%			
SBAY-F91	Ubiquitin carboxyl-terminal hydrolase 4	GB	1	1,8%			
SBAM-F23	Ubiquitin-conjugating enzyme E1	GNB	1	2,6%			
SBAU-F24	Ubiquitin-conjugating enzyme E2-17 kDa	GNB	1	2,2%			
Contig184	Ubiquitin-conjugating enzyme E2-17 kDa 3	GB	2	2,1%	2		
Contig65	UTP-glucose-1-phosphate uridylyltransferase	GNB	1	1,3%		Contig65	2
Contig44	UTP-glucose-1-phosphate uridylyltransferase	GNB	1	1,4%		Contig65	2

SBAT-F56	UTP-glucose-1-phosphate uridylyltransferase	GB	1	2,0%			
Contig213	Vacuolar ATP synthase 16 kDa proteolipid subunit	GB	2	1,8%	2		
SBAE-F93	Vacuolar ATP synthase 16 kDa proteolipid subunit	GB	1	0,8%			
SBAF-F50	Vacuolar ATP synthase catalytic subunit A	GB	1	0,8%			
Contig167	Vacuolar ATP synthase subunit B	GNB	2	2,4%			
Contig155	Vacuolar ATP synthase subunit D	GNB	2	0,6%	3		
SBAR-F41	Vacuolar protein sorting 29	GB	1	1,2%			
SBBF-F53	Vacuolar proton-ATPase subunit, putative	GB	1	1,4%			
SBBA-F11	Valine-tRNA ligase	GB	1	2,8%			
SBBA-F80	WD-40 repeat protein	GB	1	5,4%			
SBBJ-F62	WD-40 repeat protein	GB	1	0,0%			
SBBR-F48	WD-40 repeat protein	GB	1	2,4%			
Contig454	Wos2 protein	GB	6	1,3%		Contig454	2
Contig337	Wos2 protein	GB	3	1,3%		Contig454	2
Contig100	VSP	GNB	1	17,3%		Contig100	5
Contig452	VSP	GNB	6	12,8%		Contig452	2
Contig379	VSP	GNB	3	17,0%		Contig452	2
Contig89	VSP	GNB	1	16,4%		Contig452	2
Contig43	VSP	GNB	1	15,3%		Contig99	2
Contig72	VSP	GNB	1	15,9%			
SBAV-F24	VSP	GNB	1	16,0%			
Contig249	VSP	GNB	2	13,4%			
Contig280	VSP	GNB	2	15,6%			
SBAF-F35	VSP	GB	1	13,4%			
Contig74	VSP with INR	GNB	1	11,4%		Contig191	2
Contig164	VSP with INR	GB	2	14,9%		Contig452	2
Contig105	VSP with INR	GNB	1	17,3%		Contig526	5
Contig77	Xaa-Pro dipeptidase	GNB	1	2,1%		Contig77	2
Contig14	Xaa-Pro dipeptidase	GNB	1	1,7%		Contig77	2
Contig143	Xaa-Pro dipeptidase	GB	2	2,8%	2		
SBBH-F22	Zinc finger domain	GNB	1	4,6%			
SBBU-F13	Zinc finger domain	GB	1	3,7%			
Contig346	Zinc finger domain	GB	3	2,6%			
SBAP-F93	Zinc finger domain	GB	1	6,0%			
SBBU-F30	Zinc finger domain	GB	1	6,6%			
SBBY-F5	Zinc finger domain	GB	1	0,0%			
SBCA-F61	Zinc finger protein	GNB	1	9,1%			
SBBR-F78	Zinc finger protein	GB	1	14,2%			

[a]) Indicates if the highest similar sequence is a homologous *Giardia* protein (*Giardia* best - GB), a homolog is present in *Giardia*, but with proteins with higher similarity outside diplomonads (Giardia non-best - GNB), or if no *Giardia* protein is among the detected homologs (*Spironucleus* unique – SU).
