# Supplementary material for: Large genomic differences between the morphologically indistinguishable diplomonads Spironucleus barkhanus and Spironucleus salmonicida
Source: BMC Genomics. 2010 Apr 21;11:258. doi: 10.1186/1471-2164-11-258 (PMC2874811; doi:10.1186/1471-2164-11-258)
Supplement: Additional file 6 — Clustered ESTs without homologs in the G. intestinalis genome. A table listing all S. barkhanus ESTs with significant sequence similarity to proteins in the public databases, but without homologs in G. intestinalis. The E values and taxonomic designation are given for the most similar sequences. [file 1471-2164-11-258-S6.PDF]

Additional file 6 - Roxström-Lindquist, *et al.*

Clustered ESTs without homologs in the *G. intestinalis* genome.

**Contig**	**annotation**	**#reads**	**species[a]**	**classification[a]**		**E[a]**
Contig393	40S ribosomal protein S19	4	*Mastigamoeba balamuthi*	*E*	*Pelobiontida*	2E-23
Contig343	Actophorin	3	*Acanthamoeba castellanii*	*E*	*Acanthamoebidae*	3E-25
Contig153[b]	Aldose 1-epimerase	2	*Acaryochloris marina*	*B*	*Cyanobacteria*	3E-16
Contig382	Aminopeptidase I	3	*Clostridium cellulolyticum*	*B*	*Firmicutes*	5E-45
SBAZ-F44	Aminotransferase	1	*Clostridium phytofermentans*	*B*	*Firmicutes*	9E-19
Contig145	Aspartyl aminopeptidase	2	*Monodelphis domestica*	*E*	*Metazoa*	4E-24
Contig83	ATP-dependent RNA helicase	1	*Bordetella avium 197N*	*B*	*β-proteobacteria*	2E-15
Contig97	Carotenoid isomerase	1	*Campylobacter lari*	*B*	*ε-proteobacteria*	5E-19
Contig177	Carotenoid isomerase	2	*Helicobacter hepaticus*	*B*	*ε-proteobacteria*	9E-46
Contig136	Conserved hypothertical protein	2	*Victivallis vadensis*	*B*	*Chlamydiae/ Verrucomicrobia group*	8E-07
Contig344	Conserved hypothertical protein	3	*Bacillus* sp. B14905	*B*	*Firmicutes*	2E-47
Contig395	Conserved hypothertical protein	4	*Lysinibacillus sphaericus*	*B*	*Firmicutes*	6E-48
SBBG-F68	Conserved hypothertical protein	1	*Xanthomonas axonopodis*	*B*	*γ-proteobacteria*	3E-07
Contig28	Conserved hypothertical protein	1	*Monosiga brevicollis*	*E*	*Choanoflagellida*	9E-08
Contig130	Conserved hypothertical protein	2	*Monosiga brevicollis*	*E*	*Choanoflagellida*	3E-06
SBAX-F16	Conserved hypothertical protein	1	*Monosiga brevicollis*	*E*	*Choanoflagellida*	3E-06
SBAR-F54	Conserved hypothertical protein	1	*Paramecium tetraurelia*	*E*	*Ciliophora*	2E-09
SBBH-F14	Conserved hypothertical protein	1	*Paramecium tetraurelia*	*E*	*Ciliophora*	1E-08
SBBL-F11	Conserved hypothertical protein	1	*Paramecium tetraurelia*	*E*	*Ciliophora*	2E-10
SBBN-F35	Conserved hypothertical protein	1	*Paramecium tetraurelia*	*E*	*Ciliophora*	2E-06
Contig216	Conserved hypothertical protein	2	*Tetrahymena thermophila*	*E*	*Ciliophora*	4E-06
SBAJ-F74	Conserved hypothertical protein	1	*Tetrahymena thermophila*	*E*	*Ciliophora*	5E-14
SBAU-F80	Conserved hypothertical protein	1	*Tetrahymena thermophila*	*E*	*Ciliophora*	2E-06
Contig347	Conserved hypothertical protein	3	*Coprinopsis cinerea*	*E*	*Fungi*	5E-12
Contig235	Conserved hypothertical protein	2	*Sclerotinia sclerotiorum*	*E*	*Fungi*	3E-06
SBAQ-F87	Conserved hypothertical protein	1	*Yarrowia lipolytica*	*E*	*Fungi*	4E-13
SBAD-F94	Conserved hypothertical protein	1	*Seculamonas ecuadoriensis*	*E*	*Jakobidae*	2E-09
Contig163	Conserved hypothertical protein	2	*Seculamonas ecuadoriensis*	*E*	*Jakobidae*	4E-15
SBBL-F84	Conserved hypothertical protein	1	*Leishmania infantum*	*E*	*Kinetoplastida*	6E-14
SBBY-F20	Conserved hypothertical protein	1	*Apis mellifera*	*E*	*Metazoa*	5E-22
SBAY-F56	Conserved hypothertical protein	1	*Mus musculus*	*E*	*Metazoa*	5E-08
Contig149	Conserved hypothertical protein	2	*Mus musculus*	*E*	*Metazoa*	7E-07
SBAZ-F12[b]	Conserved hypothertical protein	1	*Nematostella vectensis*	*E*	*Metazoa*	6E-09
Contig220[b]	Conserved hypothertical protein	2	*Ornithorhynchus anatinus*	*E*	*Metazoa*	6E-10
Contig361[b]	Conserved hypothertical protein	3	*Strongylocentrotus purpuratus*	*E*	*Metazoa*	6E-06
SBBV-F69	Conserved hypothertical protein	1	*Physarum polycephalum*	*E*	*Mycetozoa*	2E-07
SBBY-F39	Conserved hypothertical protein	1	*Trichomonas vaginalis*	*E*	*Parabasalidea*	8E-07
SBBK-F47[b]	Conserved hypothertical protein	1	*Blastocystis hominis*	*E*	*Stramenopiles*	2E-22
Contig241	Conserved hypothertical protein	2	*Blastocystis hominis*	*E*	*Stramenopiles*	3E-07
SBBU-F84	Conserved hypothertical protein	1	*Arabidopsis thaliana*	*E*	*Streptophyta*	2E-07
SBBU-F89	Conserved hypothertical protein	1	*Physcomitrella patens*	*E*	*Streptophyta*	1E-07
Contig483	Desulfoferrodoxin	8	*Methanococcoides burtonii*	*A*	*Euryarchaeota*	1E-09
SBBN-F58	DNA-directed RNA polymerase subunit H	1	*Thermococcus kodakarensis*	*A*	*Euryarchaeota*	7E-06
SBBU-F5	dTDP-D-glucose 4,6-dehydratase	1	*Entamoeba dispar*	*E*	*Entamoebidae*	8E-17
Contig439	Fructokinase	5	*Parabacteroides merdae*	*B*	*Bacteroidetes*	7E-14
SBAZ-F8	Histone H3 methyltransferase complex	1	*Physcomitrella patens*	*E*	*Streptophyta*	6E-10
Contig255	Nitroreductase	2	*Entamoeba histolytica*	*E*	*Entamoebidae*	8E-15
SBAH-F81[b]	Palmitoyl-protein thioesterase 3	1	*Jakoba libera*	*E*	*Jakobidae*	1E-21
Contig183	Palmitoyl-protein thioesterase 3	2	*Dictyostelium discoideum*	*E*	*Mycetozoa*	5E-22
Contig157[b]	Peptidase T	2	*Finegoldia magna*	*B*	*Firmicutes*	4E-28

SBBH-F70[b]	Peptidase T	1	*Naegleria gruberi*	*E*	*Heterolobosea*	7E-13
SBBP-F33[b]	Putative glucokinase, ROK family protein	1	*Caldicellulosiruptor saccharolyticus*	*B*	*Firmicutes*	2E-23
Contig46	Repetitive protein	1	*Bordetella avium 197N*	*B*	*β-proteobacteria*	4E-12
Contig125	Repetitive protein	2	*Bordetella avium 197N*	*B*	*β-proteobacteria*	6E-12
Contig58	Rubrerythrin	1	*Parabacteroides distasonis*	*B*	*Bacteroidetes*	3E-42
Contig427[b]	Rubrerythrin	5	*Geobacter sulfurreducens*	*B*	*δ-proteobacteria*	3E-49
Contig564	Rubrerythrin	27	*Geobacter sulfurreducens*	*B*	*δ-proteobacteria*	1E-49
SBBC-F56	Selenophosphate synthetase	1	*Haemophilus ducreyi*	*B*	*γ-proteobacteria*	6E-35

[a]) Indicate the species, classification (E=Eukaryote, B=Bacteria) and E value for the best matches in similarity searches.
[b]) Indicate contigs which have sequence similarity to *S. salmonicida* genes of putative lateral transfer origin [1].

1. Andersson JO, Sjögren ÅM, Horner DS, Murphy CA, Dyal PL, Svärd SG, Logsdon Jr JM, Ragan MA, Hirt RP, Roger AJ: **A genomic survey of the fish parasite *Spironucleus salmonicida* indicates genomic plasticity among diplomonads and significant lateral gene transfer in eukaryote genome evolution.** *BMC Genomics* 2007, **8:**51.
