# Supplementary material for: Large genomic differences between the morphologically indistinguishable diplomonads Spironucleus barkhanus and Spironucleus salmonicida
Source: BMC Genomics. 2010 Apr 21;11:258. doi: 10.1186/1471-2164-11-258 (PMC2874811; doi:10.1186/1471-2164-11-258)
Supplement: Additional file 7 — S. barkhanus pyruvate kinase alleles. An alignment of individual alleles of S. barkhanus pyruvate kinase identified in the PCR experiments. [file 1471-2164-11-258-S7.PDF]

Additional file 7 - Roxström-Lindquist, *et al.*

Alignment of identified alleles for the pyruvate kinase gene.

Numbers in parentheses indicate number of sequenced clones of the allele. Bold and normal fonts indicate major and minor alleles, respectively.

```
A1 (43)   TCCCACGGTACCTGTGCTGACCACACCGAATATTTCAACAACATCAAGGAAGGCGAAAAGATTGCCGGGCGTAATGTCCACATCTTTGGTGATATCCAGG 100
A2 (8)    ..............................................................C.....................................
A3 (6)    ..T.................................................................................................
A4 (2)    ....................................................................................................
A5 (2)    ..T.................................................................................................
A6 (2)    ....................................................................................................
A7 (1)    ..T.................................................................................................
A8 (1)    ..T.................................................................................................
A9 (1)    ..............................................................C.....................................
A10 (1)   ....................................................................................................
A11 (1)   ..T...........................................................C.....................................

A1 (43)   GCCCCAAGCTCAGAATCAACAAGTTCGCCGGCGGACCACAGCAGGTCGTCACTGGTCAGTCTTTCACTCTCGACGAGTCACCAGTCGATGGTGATAACAC 200
A2 (8)    .......................................................A................................C...........
A3 (6)    ..................................................................TC....................C...........
A4 (2)    .......................................................A................................C...........
A5 (2)    ....................................................................................................
A6 (2)    ..................................................................TC....................C...........
A7 (1)    ..................................................................TC....................C...........
A8 (1)    ..................................................................TC....................C...........
A9 (1)    ....................................................................................................
A10 (1)   ..................................................................TC....................C...........
A11 (1)   .......................................................A................................C...........

A1 (43)   CCGTGTTTACCTTCCCCACCCCGAGTTCTTCGACGTCTGCCAGGTTGGTGACGCCATCCTCATCAATGACGGTATCGTCGTAGTTGAATGCACCAAGAAC 300
A2 (8)    ....................................................................................................
A3 (6)    ...................................................T................................................
A4 (2)    ....................................................................................................
A5 (2)    ....................................................................................................
A6 (2)    ...................................................T................................................
A7 (1)    ...................................................T................................................
A8 (1)    ...................................................T................................................
A9 (1)    ....................................................................................................
A10 (1)   ...................................................T................................................
A11 (1)   ....................................................................................................

A1 (43)   CACACTAACGCCGCTGGTATCAGAGAAATCGTCACCAAGGTCGTCCGTGAGGGTACCATCTCTGACAGAAAGGGTGTCGCTCTCCCAGCTCGTGTTCTCC 400
A2 (8)    .....C........................................................C...................................T.
A3 (6)    .....C........................................................C...................................T.
A4 (2)    .....C........................................................C...................................T.
A5 (2)    ....................................................................................................
A6 (2)    .....C........................................................C...................................T.
A7 (1)    .....C........................................................C...................................T.
A8 (1)    .....C........................................................C...................................T.
A9 (1)    ....................................................................................................
A10 (1)   .....C..............................................................................................
A11 (1)   .....C........................................................C...................................T.

A1 (43)   CCCTGAGCACCACCTCTCCTCGTGATATCGAATGCATCGAGAACGCCTGCAACCTCGGTATCGAGT 466
A2 (8)    ................C.................................................
A3 (6)    .A..............C................................T................
A4 (2)    ................C.................................................
A5 (2)    ..................................................................
A6 (2)    .A..............C................................T................
A7 (1)    ................C................................T................
A8 (1)    .A................................................................
A9 (1)    ..................................................................
A10 (1)   ..................................................................
A11 (1)   ................C.................................................
```
