# Supplementary material for: Large genomic differences between the morphologically indistinguishable diplomonads Spironucleus barkhanus and Spironucleus salmonicida
Source: BMC Genomics. 2010 Apr 21;11:258. doi: 10.1186/1471-2164-11-258 (PMC2874811; doi:10.1186/1471-2164-11-258)
Supplement: Additional file 8 — PFGE analyses of S. barkhanus and S. salmonicida chromosomal DNA. The results of the PFGE experiments together with densitometry analyses and a discussion of the results. [file 1471-2164-11-258-S8.PDF]

Additional file 8 - Roxström-Lindquist, *et al.*

Figure S1. PFGE analyses of *S. barkhanus* and *S. salmonicida* chromosomal DNA.
(A) PFGE run at 12°C with 2.0 V/cm at 120° angle and 900 s switch time for 13 h, 1800 s for 14 h, 2700 s for 28 h, 3600 s for 28 h, and 4500 s for 28 h, and (B) PFGE run at 14°C with 2.0 V/cm at 106° angle and 120 s switch time for 12 h, 500 s for 24 h, and 1800 s for 24 h. *S. barkhanus* and *S. salmonicida* chromosomal DNAs are shown in lane 1 (A and B) and lane 4 (A) and lane 5 (B), respectively. Chromosomal DNA from *S. cerevisiae* (0.225 – 2.2 Mb, lane 3 (A) and lane 4 (B)), *H. wingei* (1.05 – 3.13 Mb, lane 2 (A) and lane 3 (B)) and *S. pombe* (3.5 – 5.7 Mb, lane 2 (B)), were used as size markers. Bands used for densitometry analysis (Table S1) are indicated by asterisks.

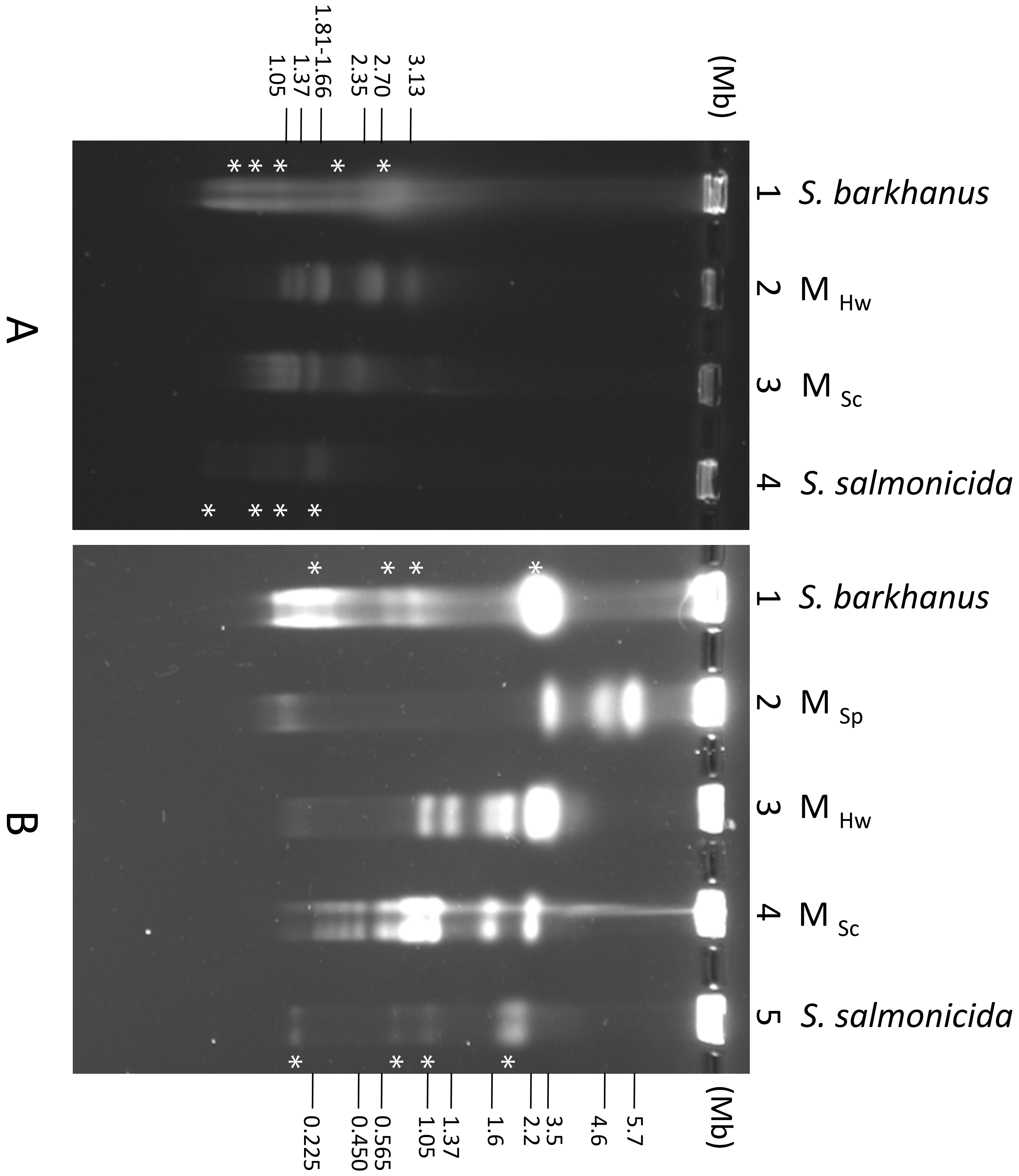

Table S1. Densitometry analysis of the PFGE analyses of *S. barkhanus* and *S. salmonicida* chromosomal DNAs.

Organism	*lane*[a]	*band*[a]	*size (Mb)*	*number of chromosomes*	*genome size (Mb)*
S. barkhanus	A1	1	2.4- 3.0	ND[b]	ND[b]
	A1	2	2.0	1	
	A1	3	0.9	1	
	A1	4	0.5	1	
	A1	5	0.2	1	
S. barkhanus	B1	1	2.0-3.5	ND[b]	ND[b]
	B1	2	0.9	1	
	B1	3	0.5	1	
	B1	4	0.2	?	
S. salmonicida	A4, B5	1	1.8-2.0	4	~9.0-9,8
	A4, B5	2	1.1	1	
	A4, B5	3	0.6	1	
	A4, B5	4	0.1	1	

[a]) Lane and band refer to Figure S1.
[b]) Not determined; the copy number of the largest bands cannot be resolved.

The chromosomal sizes range from 0.1 to 2.0 Mb for *S. salmonicida*, and 0.2 to 3.5 Mb for *S. barkhanus* (Figure S1 and Table S1). Although the band at 0.1 Mb for *S. salmonicida* is smaller than that of usual chromosomes it is included as it was repeatedly found in parallel experiments; further studies are needed to clarify its identity. We used the SynGene software to test whether any of the bands contained several unresolved chromosomes (Table S1). For *S. salmonicida* the intensity of the band that range from 1.8 to 2.0 Mb points to the presence of four chromosomes (Figure S1B, lane 5 and Table S1). If so, the electrophoretic karyotype of *S. salmonicida* corresponds to seven chromosomes (0.1, 0.6, 1.1, and four with sizes between 1.8 and 2.0 Mb) with an estimated haploid genome size between 9.0 and 9.8 Mb (Table S1). For *S. barkhanus*, the PFGE experiments were inconclusive. Single bands of the sizes 0.2, 0.5 and 0.9 Mb were identified with additional bands in the 2.0 to 3.5 Mb range (Figure S1). Unfortunately, the number of the larger bands could not be resolved in any of the experiments (Table S1).
