# Supplementary material for: Large genomic differences between the morphologically indistinguishable diplomonads Spironucleus barkhanus and Spironucleus salmonicida
Source: BMC Genomics. 2010 Apr 21;11:258. doi: 10.1186/1471-2164-11-258 (PMC2874811; doi:10.1186/1471-2164-11-258)
Supplement: Additional file 9 — PCR primers for sequence heterogeneity studies. A table of PCR primers used to study sequence heterogeneity in the Spironucleus enolase, ribosomal protein S2, glutamate dehydrogenase, hsp70, and pyruvate kinase genes. [file 1471-2164-11-258-S9.PDF]

Additional file 9 - Roxström-Lindquist, *et al.*

PCR primers used to amplify regions of five genes from *S. barkhanus* and *S. salmonicida* genomic DNA to study sequence heterogeneity.

**Gene**	**primer**	**sequence**
Enolase	EnoSpiroF	CTGCCAACAAGACYTTCGGTAAGC
Enolase	EnoSpiroR	CGTCCTGRTCGAAGGGATCTTC
Ribosomal protein S2	RiboS2SpiroF	ACGGAAAGAACGTAAGAACAGCG
Ribosomal protein S2	RiboS2SpiroR	CGTCTTCAATACCWGCAAGAGC
Glutamate dehydrogenase	GDHSpiroF	GGACTWCGTTTCCACCCTTC
Glutamate dehydrogenase	GDHSpiroR	CCTCTCTTMAYGTTCTTGAGKTCC
HSP70	HSP70SpiroF	CGTCTCATYGGTGAGGCTGC
HSP70	HSP70SpiroR	CCACCAAGATGAGAGTTACCAGC
Pyruvate kinase	PyrSpiroF	CACCACCATCCGTATCAACTTC
Pyruvate kinase	PyrSpiroR	GGATGAAGGARAGCATGATGC
